# Improvement of Precision in Recombinant Adeno-Associated Virus Infectious Titer Assay with Droplet Digital PCR as an Endpoint Measurement

**DOI:** 10.1089/hum.2023.014

**Published:** 2023-08-16

**Authors:** Tam Duong, James McAllister, Khalid Eldahan, Jennifer Wang, Eric Onishi, Kate Shen, Robert Schrock, Bingnan Gu, Peng Wang

**Affiliations:** ^1^ Department of Research & Development, Lonza Houston, Inc., Houston, Texas, USA.; ^2^ Global Operations, Lonza Portsmouth, Inc., Portsmouth, New Hampshire, USA.; ^3^ Process Development and Bioassay Service, Lonza Houston, Inc., Houston, Texas, USA.

**Keywords:** AAV, infectious titer, TCID_50_, ddPCR, qPCR, infectivity scoring, threshold

## Abstract

Recombinant adeno-associated virus (rAAV) has been utilized successfully for *in vivo* gene delivery for treatment of a variety of human diseases. To sustain the growth of recombinant AAV gene therapy products, there is a critical need for the development of accurate and robust analytical methods. Fifty percent tissue culture infectious dose (TCID_50_) assay is an *in vitro* cell-based method widely used to determine AAV infectivity, and this assay is historically viewed as a challenge due to its high variability. Currently, quantitative PCR (qPCR) serves as the endpoint method to detect the amount of replicated viral genome after infection. In this study, we optimize the TCID_50_ assay by adapting endpoint detection with droplet digital PCR (ddPCR). We performed TCID_50_ assays using ATCC AAV-2 reference standard stock material across 18 independent runs. The cell lysate from TCID_50_ assay was then analyzed using both qPCR and ddPCR endpoint to allow for direct comparison between the two methods. The long-term 1-year side-by-side comparison between qPCR and ddPCR as endpoint measurement demonstrated improved interassay precision when the ddPCR method was utilized. In particular, after the addition of a novel secondary set threshold for infectivity scoring of individual wells, the average infectious titer of 18 runs is 6.45E+08 with % coefficient of variation (CV) of 42.5 and 5.63E+08 with % CV of 34.9 by qPCR and ddPCR, respectively. In this study, we offer improvements of infectious titer assay with (1) higher interassay precision by adapting ddPCR as an endpoint method without the need of standard curve preparation; (2) identification of a second “set threshold” value in infectivity scoring that improves assay precision; and (3) application of statistical analysis to identify the acceptance range of infectious titer values. Taken together, we provide an optimized TCID_50_ method with improved interassay precision that is important for rAAV infectious titer testing during process development and manufacturing.

## INTRODUCTION

In gene therapy, adeno-associated virus (AAV) is one of the leading gene delivery platforms for the treatment of human diseases. The first approved recombinant AAV (rAAV) gene therapy product by the European Medicines Agency is alipogene tiparvovec (Glybera^®^) for the treatment of lipoprotein lipase deficiency.^1^ Recently, AAV has also shown great potential in several clinical trials, including the treatment of aromatic l-amino acid decarboxylase deficiency, Parkinson's disease, achromatopsia, and hemophilia B (reviewed by Wang et al.^2^). AAV is a nonenveloped virus that can be engineered as rAAV to deliver DNA to target cells. Advantages of AAV-mediated gene transfer include high biosafety, low immunogenicity, a broad range of infectivity, and stable expression by providing long-term gene delivery *in vivo*.^2,3^

Together with the substantial growth of rAAV applications in *in vivo* gene therapy, rAAV analytical methods also need to be continuously improved and optimized to accurately characterize and fully understand rAAV products.^4^ In particular, accurate measurement of rAAV infectious titer values remains a fundamental challenge in the field due to the highly variable nature of biological cell-based assays.^5,6^ Improvements in 50% tissue culture infectious dose (TCID_50_) assay accuracy and precision will facilitate AAV stability studies, evaluation of critical quality attributes, and AAV product release.^4,7^

The TCID_50_ assay is an *in vitro* cell-based assay widely used to evaluate infectious titer of rAAV viral vector products.^4^ This assay utilizes HeLaRC32 cells, which are genetically modified HeLa cells expressing the *rep* and *cap* genes required for AAV replication and packaging.^8^ Cells are co-inoculated with serial dilutions of the AAV test article and adenovirus type 5 (Ad5) helper virus, which provide essential helper components to facilitate viral replication.^9^

AAV infection does not cause cytopathic effects *in vitro*; therefore, the current TCID_50_ assay for AAV utilizes quantitative PCR (qPCR) as an endpoint method to detect the amount of replicated viral genome after 2 days of infection.^9,10^ qPCR-based analyses evaluate the cycle threshold (C_T_) values of AAV+Ad5 co-inoculated wells relative to control wells (inoculated with Ad5 only) to identify infected wells. Together with the dilution logs used in the assay and the ratio of total replicate wells at each dilution level showing positive infection, the Spearman-Kärber method is applied to determine the amount of rAAV required to infect 50% of the wells (Materials and Methods section).^11,12^

Due to the nature of cell-based assays, the TCID_50_ assay has high variability with a coefficient of variation (CV) up to 60% between individual runs.^6,13^ For viral genome titer assay, qPCR-based method requires additional assay optimization and depends on long-term availability of standard curve materials to obtain accurate quantitative measurement.^14^ To improve the precision of the TCID_50_ assay, we aimed to adapt the endpoint analysis of this assay using the droplet digital PCR (ddPCR). The adaptation of the ddPCR method in TCID_50_ endpoint detection was mentioned previously in an analytical method review.^4^ However, there is still need for a comprehensive study regarding the application of ddPCR read-out for the analysis and calculation of AAV infectious titer. In this study, we evaluate how well the ddPCR method can be adapted to TCID_50_ assay, and how this adaptation can improve assay performance, which is critical for the measurement of rAAV quality and stability in a manufacturing environment.

ddPCR assay is a next-generation technology for highly precise and reproducible absolute quantification of the copy number of DNA/viral genomes.^15^ As an improvement over traditional qPCR methods, ddPCR uses a microfluidic device to partition a PCR into ∼20,000 individual droplets. Each of these nanoliter-sized droplets serves as a separate and individual PCR. The fluorescence of each droplet is measured and divided into a population of either positive or negative droplets based on an indicated experimental threshold value. By a combination of limiting dilution and Poisson distribution statistics, ddPCR provides an absolute measurement of concentrations without the need for a standard curve.^16^ With the application of this method to AAV viral genome titering,^17^ it has been demonstrated to improve accuracy and intra-assay and interassay precision, and is more resistant to PCR inhibitors.^14,15,18–21^

By monitoring 18 independent runs and directly comparing the read-out between the two methods, we observed a trend of improvement in assay precision when using newly adapted ddPCR compared to qPCR. Furthermore, we identified a novel secondary “set threshold” technique for infectivity scoring, which can be used to minimize scoring false positives in higher dilution wells. In addition, we optimized the TCID_50_ assay workflow without a DNA extraction step, which can further improve high throughput and consistency between individual wells in a 96-well plate format.

Taken together, we optimized the traditional TCID_50_ assay by offering the improvements of: (1) higher precision by adapting ddPCR as an endpoint method without the need of standard curve preparation; (2) identification of a second “set threshold” value in infectivity scoring that improves assay precision; and (3) application of statistical analysis to identify the acceptance range of infectious titer values.

## MATERIALS AND METHODS

### HeLaRC32 cell culture

HeLaRC32 cells (CRL-2972; ATCC) are genetically modified HeLa cells that express the *rep* and *cap* genes required for AAV replication and packaging. HeLaRC32 cells were cultured in DMEM high glucose (HG), GlutaMAX supplement media (10-566-024; Gibco) supplemented with 1% HEPES and 10% defined fetal bovine serum (SH30070.03HI; Cytiva Hyclone). Cells were maintained in a 37°C humidified incubator at 5% CO_2_. Cells were used from passage 3 to passage 12 for the TCID_50_ assays.

### Viral vector

rAAV, AAV2 reference standard material (AAV2 rss) was purchased from ATCC (Cat. No. VR-1616).^22,23^ Human adenovirus 5 (Ad5) was obtained from ATCC (Cat. No. VR-1516). Upon receipt, AAV2 and Ad5 were thawed at room temperature, aliquoted into 10 μL single-use aliquots, and stored at −80°C. All aliquoting was performed under aseptic conditions.

### Experimental design

In this assay design, we used a 96-well plate format as previously described.^9^ This plate included serial dilutions of AAV2 from D1 to D7 (viral genome concentration from 1.00E+06 to 1.00E+00 vg/mL, Supplementary Table S2 for dilution scheme) and Ad5-only negative control (NC). Each dilution and NC were replicated in 10 wells for high precision of this quantal type assay,^9^ together with 8 wells of uninfected control (UI) (Supplementary Table S1).

**Figure 1.**
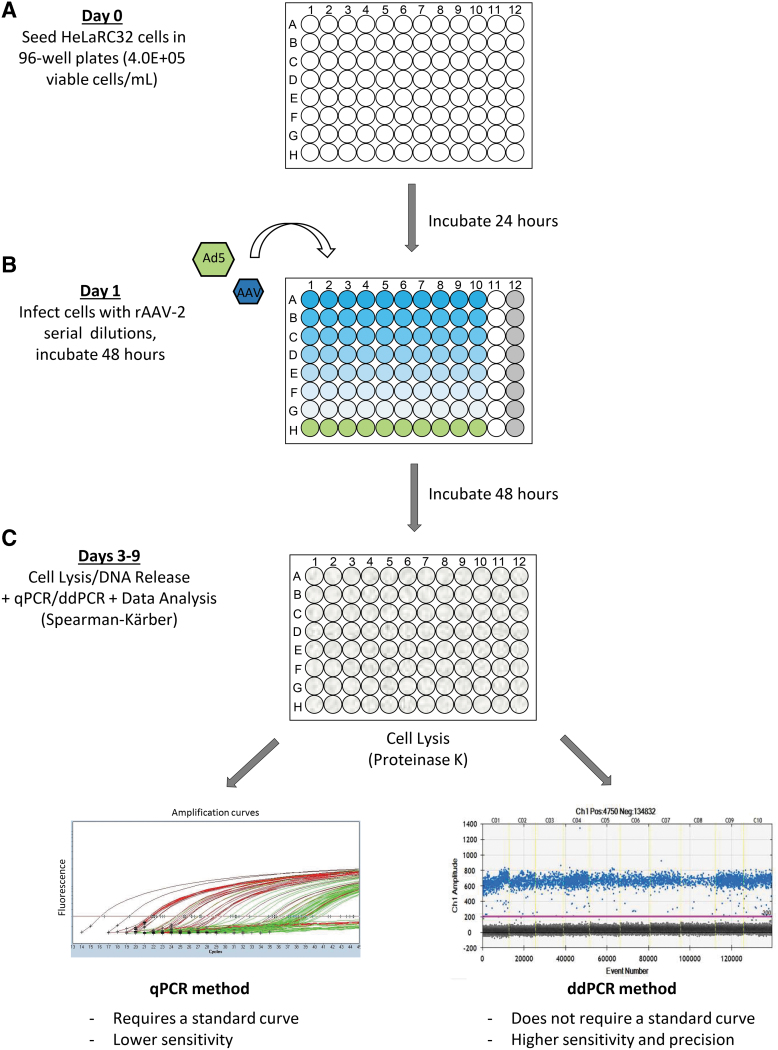
TCID_50_ workflow with qPCR and ddPCR as an endpoint measurement. TCID_50_ is a cell-based assay to measure infectious virus titer. The assay workflow is as follows: **(A)** Day 0: cell plating. HeLaRC32 cells were plated in 96-well plate at the density of 4.0E+05 cells/mL. **(B)** Day 1: infection. Twenty-four hours after cell plating, series of diluted viral vectors were added to the wells accordingly, along with Ad5 helper virus; the infection plate layout is explained in Supplementary Table S1 (gradient *blue* code is for serial dilution of AAV-2 co-infected with Ad5, *green* is for Ad5 control-only wells, and *gray* is for UI wells. **(C)** Day 3: cell lysis. Forty-eight hours after infection, cells were lysed using Proteinase K solution; Day 3–9: endpoint detection. Cell lysate was diluted 1:50 or 1:100 in TE buffer before use with ddPCR or qPCR runs. The results were then analyzed according to the Spearman-Kärber method to calculate the TCID_50_ in terms of infectious titer (in IU/mL) and specific infectivity (vg/IU). AAV-2, adeno-associated virus-2; ddPCR, droplet digital PCR; IU, infectious unit; qPCR, quantitative PCR; TCID_50_, 50% tissue culture infective dose; TE, Tris-EDTA buffer; vg, viral genome.

Each well served as an individual test unit, which was scored as “positive” or “negative” for infection by comparing to the NC threshold. Forty-eight hours after infection, the cells were lysed and diluted at 1:50 or 1:100 in TE buffer and then used directly for qPCR or ddPCR to detect viral genome replication (Fig. 1). The same 96-well layout was used in qPCR and ddPCR plate setup, except for column 12, which was used for the standard curve and nontemplate control (NTC) (Supplementary Table S3). This similar layout between the infection plate and PCR plate was designed to facilitate high throughput, so that the same layout could be programmed for multiple samples in an automated system.

### Viral infection

HeLaRC32 cells were plated in a 96-well plate 24 h before viral infection at a density of 4.0E+05 viable cells/mL and volume of 50 μL per well for a total of 2.0E+04 viable cells/well. Cell count and cell viability were measured using a Vi-Cell Cell Viability Analyzer (Beckman Coulter). Infection media consisted of DMEM HG with GlutaMax supplemented with 1% HEPES (serum-free media). The infection media was stored at 4°C and warmed up to 37°C before use.

Vector diluent was prepared with Ad5 vector at a final concentration of 3.20E+08 vp/mL in 15 mL of serum-free DMEM supplemented with 1% HEPES (for each 96-well plate) as previously described.^22^ All AAV2 viral dilutions were prepared using this vector diluent with a static concentration of Ad5. AAV2 was serially diluted from 1.00E+06 to 1.00E+00 vg/mL for TCID_50_ assay using the same vector diluent; therefore, the Ad5 concentration stays the same at 3.20E+08 vp/mL in all AAV2 dilution group (Supplementary Table S2).

Culture media was first removed from the wells, and then 50 μL of the diluted viral vector was applied to each well according to the infection plate layout (Supplementary Table S1). The plate was incubated at 37°C in a humidified incubator at 5% CO_2_ for 2 h before the addition of 50 μL of complete culture medium. The total volume of each well was 100 μL at the end of infection step. The plate was then incubated at 37°C in a humidified incubator at 5% CO_2_ for another 48 h before cell lysis.

### Cell lysis

The cells were lysed 48 h after infection, using 10 μL Proteinase K mix (Proteinase K 20 mg/mL [MC5008; Promega] +10 × Proteinase K buffer [1% SDS, 10 mM ethylenediamine tetra-acetic acid (EDTA), 10 mM Tris pH 8.0]). The plate was incubated at 37°C for 1 h, and then the cell lysates were transferred to a 96-well PCR plate and incubated in a thermocycler (program: 55°C for 60 min to inactivate the Ad5, 95°C for 10 min to inactivate the Proteinase K, and then 2–8°C hold). Cell lysates were stored at 4°C for up to 7 days and diluted at 1:50 or 1:100 using TE buffer immediately before the PCR analysis.

### Quantitative PCR

qPCR was performed in 20 μL reactions containing 5 μL of diluted cell lysis (1:50) following the qPCR plate layout (Supplementary Table S3). PerfeCTa 2X qPCR Supermix (101414-128; Quantabio) was used to make qPCR mastermix, along with 500 nM CMV-F (forward primer), 500 nM CMV-R (reverse primer), and 250 nM CMV-probe (Supplementary Table S12). PCR thermocycler program: Stage 1 (95°C, 5 min) and Stage 2 (95°C, 15 s; 60°C, 1 min; repeat for 45 cycles).

For the standard curve, we used linearized plasmid pAAV-GFP (AAV-400; Cell Biolabs) cut with *Hin*dIII restriction enzyme (R3104; NEB). The linearized plasmid used for the standard curve was purified using Qiagen Quick PCR Purification Kit (Cat. 28104). Purified linearized plasmid was then diluted in standard diluent (40 μL of *Lambda Hin*dIII DNA [0.5 μg/μL] in 9.96 mL of 1 × TE [pH 7.4]) to make serial 10 × dilution from 2.00E+06 to 2.00E+00 copies/μL. A set of seven standards was aliquoted into an 8-strip tube and stored at −20°C. Each strip serves as a single-use standard curve for each qPCR plate. Each standard was used at 5 μL in one qPCR run.

### Droplet digital PCR

ddPCR was performed in 22 μL reactions containing 5 μL of Tris-EDTA (TE) buffer diluted cell lysate at either 1:50 dilution or 1:100 dilution following the plate layout (Supplementary Table S3). 2 × ddPCR Supermix for Probes (1863010; Bio-Rad) was used in the ddPCR together with 900 nM CMV-F primer, 900 nM CMV-R primer, and 62.5 nM CMV-probe. Droplets were generated with the QX200 Droplet Generator following the manufacturer's Bio-Rad protocol. In this system, samples were partitioned into ∼20,000 nanoliter-sized droplets. PCR amplification was then performed with Bio-Rad C1000 Touch Thermo Cycler: Stage 1 (95°C, 10 min); Stage 2 (94°C, 30 min; 56°C, 1 min; repeat for 40 cycles); Stage 3 (98°C, 10 min); and Stage 4 (4°C, hold), with temperature ramp at 2°C/s. Droplets were read using the QX200 Droplet Reader and results were analyzed with QuantaSoft software.

### TCID_50_ calculation

Each dilution level was scored in 10 replicate wells for positive infection by comparison to the threshold values derived from Ad5-only control wells. *C*_T_ values were used for qPCR-based scoring (Table 1), while copies/μL values were used for ddPCR-based scoring (Table 2). Individual wells that scored positive for infection were assigned a value of 0.1 and negative wells were assigned a value of 0.0. The sum of ratios of infectious positive wells was calculated based on the total assigned value of 10 replicates at each dilution. Using the dilution logarithms in the assay and the ratio of total positive infection wells at each dilution level, the Spearman-Kärber method was then applied to determine the amount of AAV test articles required to infect 50% of wells. Below is the formula to calculate the infectious titer in infectious units per milliliter (IU/mL):

**Table 1.** Interpretation of quantitative PCR results for TCID_50_ analysis of adeno-associated virus-2 rss

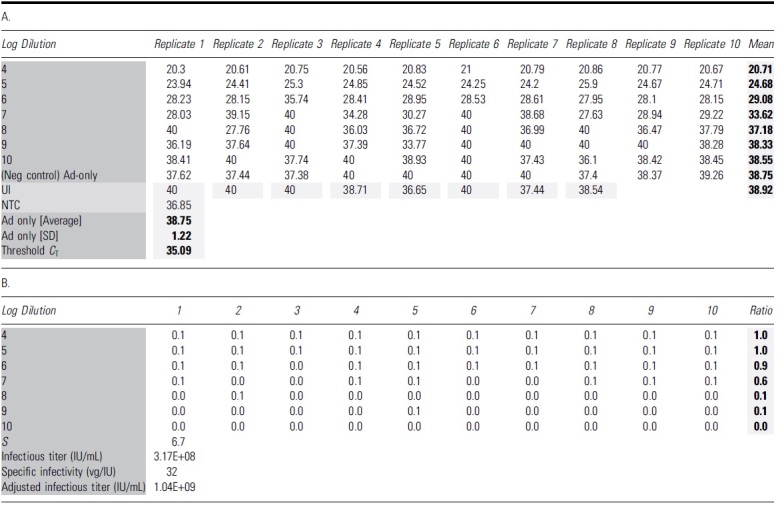

The bold format of number values is to highlighted the calculated value of Mean, Ad5 avg, SD and Threshold.

The dark and light grey shadings are to highlight the table's name groups and Mean and calculated average/SD and Threshold value.

(A) The table shows all *C*_T_ values of 96 individual wells with qPCRs using 1:50 diluted cell lysate. The average *C*_T_ value for Ad5-only infected wells was 38.75 with an SD of 1.22. Threshold *C*_T_ for this run is calculated as Ad5-only mean minus three SD (35.09). (B) Wells that yielded *C*_T_ values lower than the identified threshold are considered positive for AAV infection, with a score value of 0.1 for each well. The ratio is identified as the total scoring value of 10 replicates for each dilution. The Spearman-Kärber method is then applied to determine the infectious titer (IU/mL) and specific infectivity (vg/IU).

*C*_T_, cycle threshold; NTC, nontemplate control; qPCR, quantitative PCR; SD, standard deviation; TCID_50_, 50% tissue culture infectious dose; UI, uninfected control.

**Table 2.** Interpretation of droplet digital PCR results for the TCID_50_ analysis of adeno-associated virus-2 rss

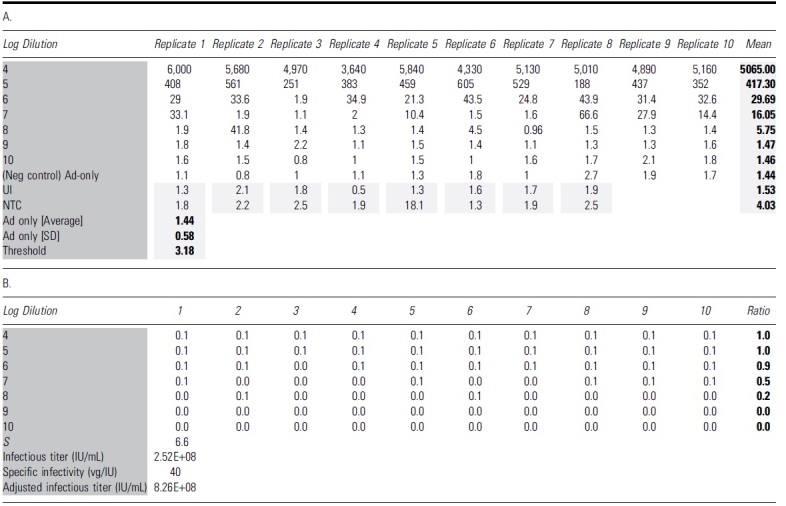

The bold format of number values is to highlighted the calculated value of Mean, Ad5 avg, SD and Threshold.

The dark and light grey shadings are to highlight the table's name groups and Mean and calculated average/SD and Threshold value.

(A) The table shows all the concentration values (vg/μL) for 96 individual wells with ddPCRs. The average copy number for the negative control Ad5-only infected wells was 1.44 with an SD of 0.58. The threshold copy number for this run was calculated as Ad5-only average plus three SD (3.18). (B) Wells that yielded copy number higher than the identified threshold (3.18) were considered positive for AAV infection with the score of 0.1 for each well. The ratio is identified as the total scoring value of 10 replicates of each dilution. The Spearman-Kärber method is then applied to determine infectious titer (IU/mL) and specific infectivity (vg/IU).

$$InfectiousTiter\left(IU∕mL\right)=10^{\left[1+S-0.5\right]}∕V$$

where

1 = logarithm (log_10_) of the lowest dilution factor where all the replicates are positive

*S* = logarithm of the initial dilution plus the sum of ratios of infectious positive wells per total wells at each subsequent dilution

0.5 = 50% of the logarithm of the lowest dilution factor

*V* = volume of the diluted test article in mL

Formula to calculate the specific infectivity as a ratio of vector genomes (vg) to infectious units (IU) (vg/IU):

$$SpecificInfectivityoftestarticle\left[vg∕IU\right]=VectorGenomeTiter\left[vg∕mL\right]∕InfectiousTiter\left[IU∕mL\right]$$

### Assay acceptance criteria

For the qPCR method, the qPCR efficiency for the standard curve must be 100% ± 15%, with standard curve slope ranging from −3.65 to −3.17. qPCR efficiency is calculated using the following formula: qPCR efficiency [%] = [10^(−1/slope)^−1] × 100. The coefficient of determination (*R*^2^) for the standard curve must be ≥0.98. The standard curve must also consist of seven standard points.

Assay control: the lowest dilution (D1) must be 100% positive and the highest dilution (D7) must not have any infectious event in all 10 replicates (infectious event is scored using both original Threshold and indicated second set Threshold).

### Statistical analysis

To determine reasonable upper and lower limits, we calculated “tolerance limits” or a “tolerance interval.” The values are calculated to include 99% of the population at a 95% confidence level. In other words, we are 95% confident that the calculated limits contain 99% of the population of interest.

Tolerance intervals were calculated for the TCID_50_ using a set of 18 measurements from 18 independent runs analyzed with 4 different threshold methods: qPCR_1 (without set threshold), qPCR_2 (set threshold as 35 *C*_T_), ddPCR_1 (without set threshold), and ddPCR_2 (set threshold as 1.64). Due to the nature of the data (*i.e.*, very wide range in reported values), the calculated lower limits are less than zero and unrealistic. To accommodate the wide range, a logarithmic transformation is used to re-scale the reported data (Supplementary Table S8). We calculated the tolerance limits on the logarithmically transformed data as detailed above and then exponentiated the limits back to the untransformed units in the original scale.

## RESULTS

### Determination of the limit of detection for ddPCR

**Figure 2.**
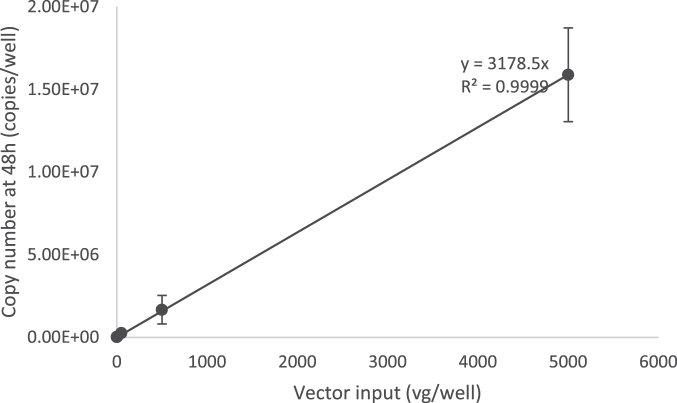
Identification of AAV-2 viral genome replication in the limiting dilution using the ddPCR method. HelaRC32 cells were infected with 10-fold serial dilutions of AAV-2 rss along with Ad5 in 96-well plate format. Vector input amounts were 0.05, 0.5, 5, 50, 500, and 5,000 viral genomes per well (vg/well). Cells were lysed with Proteinase K mix, and 1/20 of diluted cell lysate (1:50) was used for ddPCR with the CMV primers and probe set to detect viral genome replication. A graph representing the input vector genome and replicated genome during infection provided a copy number linear response with an identified replication rate at 3,178 copies/vg (*R*^2^ = 0.99, slope = 3,178 copies/vg) (n = 8 independent runs).

According to the Bio-Rad droplet digital application guide, the recommended dynamic range of the QX200 system is from 1 to 120,000 genome copies/20 μL reaction. In the previously described AAV infectious titer TCID_50_ method using qPCR detection,^9^ AAV2 vector genomes were identified to be replicated at ∼2,670-fold in 48 h with nonlimiting adenovirus co-infection. Using ddPCR endpoint detection, we calculated that the AAV2 reference standard stock material (AAV2 rss) replication rate was ∼3,178-fold in 48 h, measured over an input range from 0.05 to 5,000 vector genomes per well (Fig. 2).

With this ddPCR as an endpoint method, when a single infectious event occurs, ∼3,178 copies of the transgene will present in that particular well after 48 h of infection (total volume of about 100 μL per well). If the cell lysate was diluted 1:50, it is estimated that there will be 0.64 copies/μL in final dilution and the use of 5 μL in a PCR will contain ∼3.18 copies/reaction.

We also checked the limit of detection for the TCID_50_ assay with a ddPCR read-out, by diluting the cell lysate further to 1:100. With this dilution, ∼1.59 copies/reaction will be detected for a single infectious event. ddPCR has a smaller detection range and is more sensitive compared to qPCR; therefore, we observed high to oversaturated signal of positive droplets from the lowest viral dilution D1 using the ddPCR method with cell lysate dilution at 1:50 (Supplementary Table S4).

We observed some differences in infectious titer (6.56E+08 and 8.26E+08 IU/mL) of the same sample run when cell lysates were diluted at either 1:50 or 1:100, respectively (Supplementary Tables S4 and S5). Taking into consideration the sensitivity of lower end of the detection range, we prefer to use a 1:50 dilution of cell lysate for both the qPCR and ddPCR methods, as this dilution is sufficient to overcome potential PCR inhibitors presenting in the cell lysate.

### Infectious titer calculation with qPCR and ddPCR endpoint method

With qPCR as an endpoint method, we compared the PCR *C*_T_ of each well with *C*_T_ measurements from 10 NC wells infected with Ad5 only. The inoculated wells with a *C*_T_ value less than the average NC (Ad5 only) minus 3 standard deviations based on 10 replicates (Ad5 only mean − 3 × SD [standard deviation]) will be scored as “positive” for infection, similar to approach described previously.^9^

**Figure 3.**
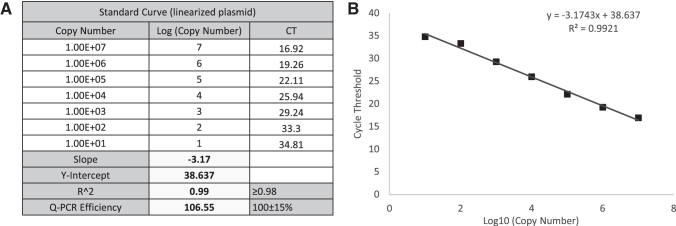
qPCR standard curve for TCID_50_ analysis of AAV-2 rss. **(A)** The table shows the serial dilutions of linearized plasmid used for the standard curve. Seven standard points were used with the copy number of linearized plasmid, from 1.00E+07 to 1.00E+01. Standard curve slope, Y-Intercept, *R*^2^, and qPCR efficiency were calculated. **(B)** The standard curve graph indicates the cycle threshold corresponding to the log (copy number) of seven standard points used. This standard curve is from one single run that represents our 18 monitored runs.

A representative scoring table and calculation of a TCID_50_ assay of AAV2 rss are provided in Table 1, including the standard curve (Fig. 3), *C*_T_ values in all infected and NC wells (“A” in Table 1), and infectivity scoring table (“B” in Table 1). In the infectivity scoring table, positive well was counted as 0.1 and negative was counted as 0.0 value. The sum of ratios of infectious positive wells was calculated based on the total counted value of 10 replicates in each dilution. Infectious titer was calculated using the Spearman-Kärber equation (Materials and Methods section) and reported in Infectious Titer (IU/mL) and in Specific Infectivity (vg/IU) (“B” in Table 1).

In the ddPCR method, we performed ddPCR endpoint analysis with the same cell lysate used for qPCR run. For the infectivity analysis, we compared the concentration (copies/μL) of each inoculated well with the average concentration from 10 NC wells that are infected with Ad5 only. The infected well with a concentration higher than the average value measured for NC (Ad5 only) plus 3 standard deviations based on 10 replicates (Ad5 only mean + 3 × SD) was scored as “positive” for infection.

**Figure 4.**
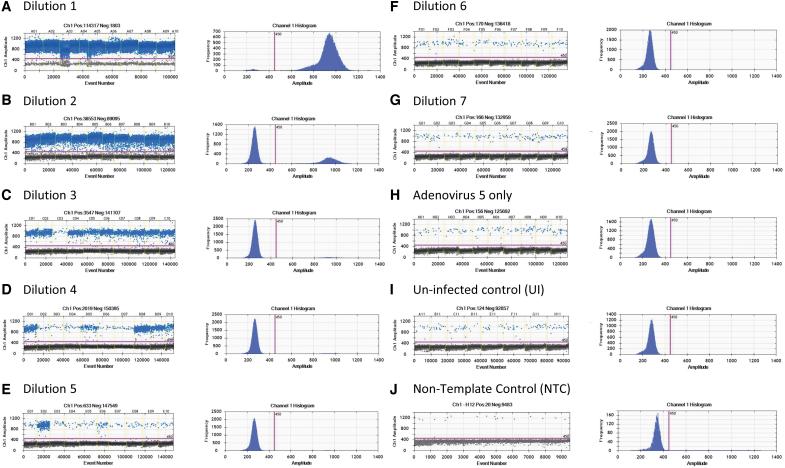
ddPCR amplitude graphs for TCID_50_ analysis of AAV-2 rss. **(A–J)** PCRs were partitioned into ∼20,000 droplets. The amplitude graphs display the separation of positive and negative droplets within 10 replicated wells of Dilution 1—D1, D2, D3, D4, D5, D6, D7, Ad5-only control, UI. and NTC. Based on the fluorescence amplitude, a single threshold line was drawn to separate the positive and negative droplets. The number of positive and negative droplets was used to calculate the viral genome concentration, using modeling as a Poisson distribution. These amplitude graphs are from one single run that represents our 18 monitored runs. NTC, nontemplate control.

We provide a representative scoring table and calculation of a TCID_50_ assay of AAV-2 rss using ddPCR as an endpoint method (Table 2), including the amplitude graphs of Ad5-only control and dilutions D1–D7, UI and NTC (Fig. 4), concentration value (“A” in Table 2), and infectivity scoring table (“B” in Table 2). Similar to the qPCR method, “positive” was counted as 0.1 and “negative” was counted as 0.0 value in the infectivity scoring table. With ddPCR as a read-out, infectious titer was calculated using the Spearman-Kärber equation and reported in Infectious Titer (IU/mL) and Specific Infectivity (vg/IU) (“B” in Table 2).

### Identification of a second “set threshold” for infectious scoring with qPCR and ddPCR

Theoretically, with qPCR as an endpoint method, the average *C*_T_ value measured for NC (Ad5 only) minus 3 times of standard deviations (based on 10 replicates) should be sufficient to serve as a threshold for infectious scoring in each well, as described in the previous AAV infectious titer method.^9^ By monitoring 18 TCID_50_ runs, we observed false positives in the highest dilution group in 2 qPCR runs and 2 ddPCR runs when the original threshold was applied into the infectious scoring method (summarized in Table 5, gray highlighted).

To distinguish between “false positive” and potential “true positive” in the highest dilution group due to operator error or cross contamination, we carefully evaluated the value from each individual and directly compared them with the experimental threshold. If the wells are true positive for infection, we should observe a notable different value compared to threshold value. We therefore identified that a second “set threshold” is necessary for infectious scoring of individual wells to avoid a potential false positive value from the highest dilution group (D7).

In one TCID_50_ run (run 11) with qPCR as an endpoint method (Supplementary Table S6), using one threshold from the average value for NC (Ad5 only) minus three standard deviations, a higher titer value was obtained due to higher infectivity scoring in the last three dilutions (D5, D6, and D7). In particular, one individual well in the highest dilution group, D7, was scored as positive and the total infectious ratios in D5, D6, and D7 groups were 0.2; 0.0; and 0.1, respectively (Supplementary Table S6B). This did not reflect the dilution scheme, in which the higher dilution group, D7, had a higher infectivity scoring compared to D6 group (Supplementary Table S6B).

In addition, the well scored positive in D7 group had a *C*_T_ value of 37.2 (Supplementary Table S6A, gray highlighted), which is higher than *C*_T_ value of the lowest concentration standard of this particular run (33.4 *C*_T_), relative to 2.0E+00 copies/μL (data not shown). *C*_T_ value of 37.21 was out of lower end of standard curve, and very close to original *C*_T_ threshold value at 37.47 (Supplementary Table S6).

In another TCID_50_ run (run 5) with ddPCR as an endpoint method (Supplementary Table S7), using one threshold from the average value for NC (Ad5 only) plus three standard deviations, a higher titer value was obtained due to higher infectivity scoring in the last three dilutions (D5, D6, and D7). In particular, one individual well in the highest dilution group D7 was scored as positive and the total infectious ratios in D5, D6, and D7 groups were 0.1; 0.2; and 0.1 respectively (Supplementary Table S7B). This did not reflect the dilution scheme, in which the higher dilution group D6 had higher infectivity scoring compared to D5 group (Supplementary Table S7B).

At D7 dilution with viral genome concentration as 1.00E+00 vg/mL, based on multiple run monitoring, we expected to observe no infectious event detected in every individual well of 10 replicates. Infectivity scoring data from four runs with false positive are also presented in Supplementary Table S13 for qPCR endpoint and Supplementary Table S14 for ddPCR endpoint detection.

To identify the second “set threshold” for this assay, 18 runs were performed to obtain the average value of the Ad5 threshold for qPCR (Table 3) and ddPCR (Table 4). This average value of Ad5 threshold was then used as a second “set threshold” for the infectious scoring. For the qPCR method, if the original threshold (Ad5 only mean − 3 × standard deviation) is higher than the “set threshold” value, then the “set threshold” will be used for the infectious scoring and vice versa.

**Table 3.** Table shows the Ad5 average and Ad5 threshold values calculated from 18 independent runs with quantitative PCR as an endpoint method

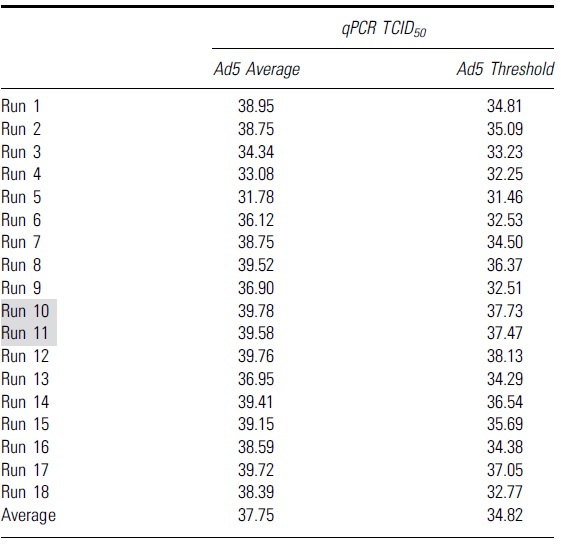

Grey highlights indicate runs that had higher infectious titers due to false positive infectious scoring in the highest dilution group, D7.

**Table 4.** Table shows the Ad5 average and Ad5 threshold values calculated from 18 independent runs with droplet digital PCR as an endpoint method

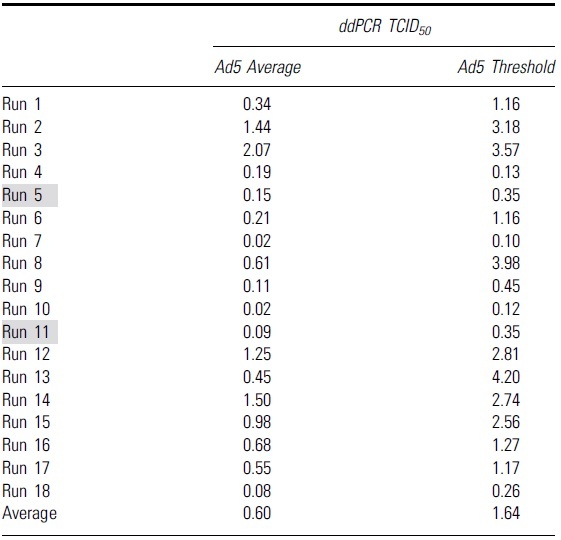

Grey highlights indicate runs that had higher infectious titers due to false positive infectious scoring in the highest dilution group, D7.

ddPCR, droplet digital PCR.

**Figure 5.**
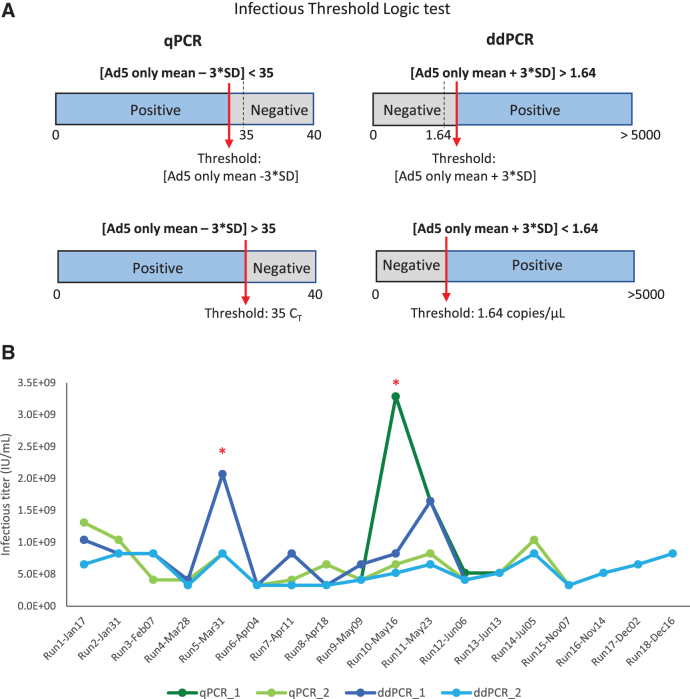
Infectious threshold logic test and 18 independent TCID_50_ runs were analyzed with qPCR and ddPCR endpoint detection. **(A)** Infectious threshold logic test. For the qPCR method, the second set threshold was identified as 35 *C*_T_. If [Ad5 only mean − 3 × SD] <35, then the [Ad5 only mean − 3 × SD] is used as threshold for infectious scoring. If [Ad5 only mean − 3 × SD] >35, then the second set threshold (35 *C*_T_) is used for infectious scoring. For the ddPCR method, the second set threshold was identified as 1.64 copies/μL. If [Ad5 only mean + 3 × SD] >1.64, then the [Ad5 only mean + 3 × SD] is used as threshold for infectious scoring. If [Ad5 only mean + 3 × SD] <1.64, then the second set threshold (1.64 copies/μL) is used for infectious scoring. **(B)** The summary graph shows infectious titer values according to 18 independent runs using different detection methods (qPCR in *green lines*, and ddPCR in *blue lines*) and different threshold methods (with and without a second set threshold). (*) Runs that had higher infectious titers due to false positive infectious scoring in the highest dilution group, D7. *C*_T_, cycle threshold; SD, standard deviation.

For the ddPCR method, if the original threshold (Ad5 only mean + 3 × standard deviation) is lower than the set threshold, then the set threshold will be used for the infectious scoring and vice versa (the infectious threshold logic test is shown in Fig. 5A). By using that average threshold value to re-score the infectious titer and re-analyze the data of each run, we got a second set of infectious titer values for both qPCR and ddPCR run (summarized in Table 5).

**Table 5.** Summary of all infectious titer values from 18 independent runs using different second set threshold with either quantitative PCR or droplet digital PCR as an endpoint detection method

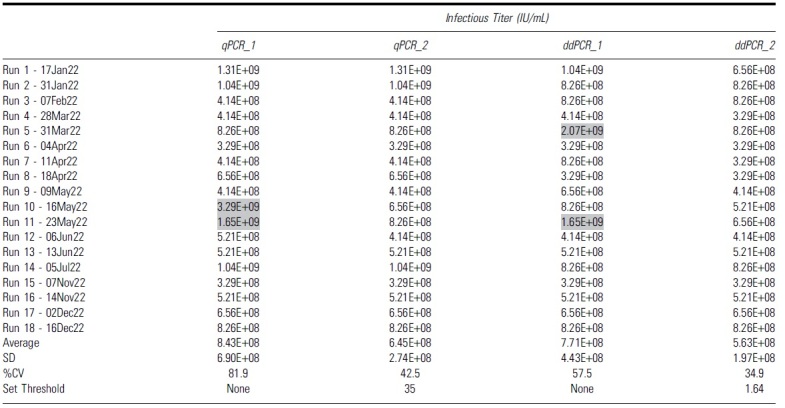

Gray highlights indicate runs that had higher infectious titers due to false positive infectious scoring in the highest dilution group, D7.

CV, coefficient of variation.

Without the second threshold gate, we observed that 2 out of 18 runs had higher infectious titers due to scored positive infectious events at, the highest dilution group, D7 (Table 5, gray highlighted). This contributed to a much higher %CV at 81.9% and 57.5% for qPCR and ddPCR, respectively (Table 5, qPCR_1 and ddPCR_1). These false positive signals that occurred in the highest dilution group may be due to low negative thresholds in some particular runs or background signals in the unpurified cell lysates. When the second threshold was used, the %CV of 18 independent runs was reduced to 42.5% and 34.9% for qPCR and ddPCR, respectively (Table 5, qPCR_2, ddPCR_2).

All the infectious titer data points using two different threshold methods either with qPCR or ddPCR as an endpoint detection are summarized in the table and graph (Table 5 and Fig. 5B). The specific infectivity (vg/IU) values are summarized in Table 6. By adding the second layer of threshold, we can eliminate false positives for individual wells at the lower ends of the dilution range (D5, D6, and D7). This might lead to more accurate infectious titer values and improve the reliability of the assay. These data indicate that the identification of a second set threshold is critical to improve precision of the TCID_50_ assay.

**Table 6.** Summary of all specific infectivity (vg/IU) values from 18 independent runs using second set threshold with either quantitative PCR or droplet digital PCR as an endpoint detection method

	ATCC AAV2-rss vg Titer: 3.28E+10 vg/mL			
	Specific Infectivity (vg/IU)			
	qPCR_1	qPCR_2	ddPCR_1	ddPCR_2
Run 1 - 17Jan22	25	25	32	50
Run 2 - 31Jan22	32	32	40	40
Run 3 - 07Feb22	79	79	40	40
Run 4 - 28Mar22	79	79	79	100
Run 5 - 31Mar22	40	40	16	40
Run 6 - 04Apr22	100	100	100	100
Run 7 - 11Apr22	79	79	40	100
Run 8 - 18Apr22	50	50	100	100
Run 9 - 09May22	79	79	50	79
Run 10 - 16May22	10	50	40	63
Run 11 - 23May22	20	40	20	50
Run 12 - 06Jun22	63	79	79	79
Run 13 - 13Jun22	63	63	63	63
Run 14 - 05Jul22	32	32	40	40
Run 15 - 07Nov22	100	100	100	100
Run 16 - 14Nov22	63	63	63	63
Run 17 - 02Dec22	50	50	50	50
Run 18 - 16Dec22	40	40	40	40
Average	56	60	55	66
SD	26	23	26	24
%CV	46.9	38.1	47.1	36.0
Set Threshold	None	35	None	1.64

In addition, to evaluate the stability of AAV2 rss over 1 year of study period, we analyzed the trending of infectious titers from 18 runs with qPCR and ddPCR method (Supplementary Fig. S1). Statistical analysis using Pearson correlation coefficient (*r*) and the test statistic *t* on the trending of infectious value over time identified no significant downward trend in infectious titer during 1 year of study. This indicated our AAV2 rss material was stable during testing period and variability observed in this study was due to TCID_50_ assay variability and not sample stability.

### Statistical analysis to identify the acceptance range of infectious titer value

**Figure 6.**
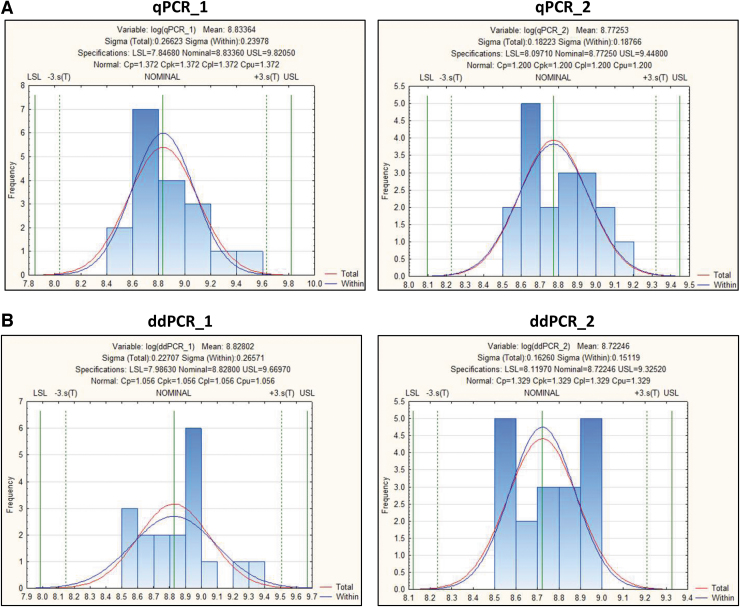
TCID_50_ statistical analysis. Tolerance intervals are calculated for the infectious titer assay using a set of 18 runs for four determinations. **(A)** qPCR_1 (no set threshold), qPCR_2 (set threshold as 35 *C*_T_), **(B)** ddPCR_1 (no set threshold), and ddPCR_2 (set threshold as 1.64 copies/μL). Due to the nature of data, a logarithmic transformation is used to re-scale the data, resulting in lower limits that will remain positive.

To further understand the acceptance range of the infectious titer of a particular AAV product, a statistical tool (STATISTICA V12/Process Capability Analysis Module) was used for the calculation of tolerance intervals. Tolerance intervals were calculated using a set of 18 measurements from 4 different methods named as qPCR_1 (no set threshold), qPCR_2 (set threshold as 35 *C*_T_), ddPCR_1 (no set threshold), and ddPCR_2 (set threshold as 1.64) (Fig. 6A, B). A logarithmic transformation was used to re-scale the data, resulting in lower limits that will remain positive and be more meaningful (Supplementary Table S8). We calculated limits that included 99% of the population at a 95% confidence interval. A summary of the calculated limits in both the log transformation and then in-transformed (exponentiated) back to the original units is identified in Table 7.

**Table 7.** Tables show Log10 transformed (A) and untransformed (B) infectious titer range from low to high values of acceptable detection ranges with the quantitative PCR and droplet digital PCR methods

A.					
	Log10 Transform				
	LSL	Nominal	USL		
qPCR_1	7.847	8.834	9.821		
qPRC_2	8.097	8.773	9.448		
ddPCR_1	7.986	8.828	9.670		
ddPCR_2	8.120	8.722	9.325		

B.					
	Untransformed/Original Units			Reported Results	
	LSL	Nominal	USL	Minimum	Maximum
qPCR_1	7.03E+07	6.82E+08	6.61E+09	3.29E+08	3.29E+09
qPCR_2	1.25E+08	5.92E+08	2.81E+09	3.29E+08	1.31E+09
ddPCR_1	9.69E+07	6.73E+08	4.67E+09	3.29E+08	2.07E+09
ddPCR_2	1.32E+08	5.28E+08	2.11E+09	3.29E+08	8.26E+08

LSL, lower specification limit; USL, upper specification limit.

Comparing the calculated limits to existing data shows that for each method, the range between the lower and upper limit is wider than the range between the minimum and maximum values for the 18 observations (Table 7), allowing us to encompass the expected variation from a cell-based assay. This indicates that the calculated tolerance intervals are reasonable and wide enough to encompass the expected variation, as illustrated in the distribution plots that follow in this analysis (Fig. 6 and Supplementary Table S11).

Further analysis was performed on logarithmically transformed data, including (1) checking for potential outliers, (2) checking for normal distribution, and (3) calculations of tolerance intervals. For potential outliers, we used the Grubbs Test in STATISTICA V12. For all four test methods, the *p*-values were above 0.05, indicating that there was no potential outlier in any of the log-transformed data (Supplementary Table S9).

To check for normal distribution, for each log-transformed variable (qPCR_1, qPCR_2, ddPCR_1, and ddPCR_2), the normal distribution is considered suitable for use. As shown in Supplementary Table S10, the *p*-values for the Kolmogorov–Smirnov test are “n.s.” or “nonsignificant” for the normal distribution (as well as other distributions). If the *p*-value is nonsignificant, we do not have enough evidence to conclude that the particular distribution does not fit the data. Since the normal distribution is suitable and no outlier is identified, the calculation for tolerance intervals can be applied (Supplementary Table S11 and Fig. 6). Using this analysis, we observed tolerance intervals of qPCR_2 (with “set threshold”) from 1.25E+08 to 2.81E+09 (IU/mL), which is similar to the ddPCR_2 range (with “set threshold”) from 1.32E+08 to 2.11E+09 (IU/mL) (Table 7).

## DISCUSSION

rAAV has been a viral vector of choice for *in vivo* gene delivery due to its high biosafety, low immunogenicity, a broad range of *in vivo* infectivity/gene delivery, and stable expression. Together with its growing demand in the field of gene therapy, the analytical aspects of rAAV manufacturing also need to be continuously improved and optimized to accurately characterize and fully understand the rAAV product. The cell-based AAV infectious titer assay (TCID_50_) remains a challenging assay to develop due to the inherently high variability of biological assays.^6,13,22^ To improve upon established AAV TCID_50_ assays,^9,10^ we have adapted a novel ddPCR-based method to replace the traditional qPCR method.

We identified a second “set threshold” for infectivity scoring, which offers higher precision and lower variability between infectious titer measurements. When the second “set threshold” was applied, there was a reduction of %CV between 18 independent runs from 81.9% to 42.5% in qPCR method and from 57.5% to 34.9% in ddPCR method. Between the two endpoint methods, we observed a trend of improved interassay precision when the ddPCR method was utilized either with or without second set threshold application. Without the addition of second threshold, the %CV of ddPCR method was 57.5%, which is lowered compared to qPCR method with a %CV of 81.9% from 18 individual runs monitored (Table 5). With the addition of second threshold, the %CV of ddPCR method was lowered to 34.9% compared to qPCR method with a %CV of 42.5% from 18 individual runs monitored (Table 5).

In general, TCID_50_ cell-based assay has high variability with a %CV up to 60% between individual runs.^6,13^ Notably, in the reference article characterizing the AAV2 rss material used in our study,^22^ the reported results for TCID_50_ infectious titer assay indicated that the %CV from multiple runs of 16 laboratories was 95.7%, and the %CV from three replicates within the same laboratory (“N”) was 73.6%. With existing TCID_50_ method, the high %CV may obscure any trend or change to infectivity resulting from process changes. The reduction we observed in the %CV using a ddPCR endpoint may therefore allow a more precise understanding of infectivity during process development and optimization, as well as during batch release.

In addition, ddPCR is an absolute quantitative method that directly measures DNA concentration (copies/μL) without reliance upon a standard curve. This makes ddPCR a more attractive method, particularly in a Quality Control setting where it streamlines assay performance and eliminates the need for system suitability acceptance criteria associated with standard curve parameters. Therefore, these factors make ddPCR easier to transfer into the highly regulated GMP/QC environment (Good Manufacturing Practice/Quality control).

Furthermore, the ddPCR method utilizes partitioned droplets as independent PCR events, which improve sensitivity, precision, and the signal-to-noise ratio, while additionally reducing the background signal of Ad5-only NCs. These improvements are crucial for the successful identification of the infectivity of a gene therapy product. We observed a very low detected signal from the Ad5-only NC group with an average of 0.02 copies/μL in two TCID_50_ runs (run 7 and 10) using ddPCR as an endpoint method (Table 4).

Notably, ddPCR can detect as low as 1 copy per 20 μL reaction, which is equal to ∼0.05 copies/μL concentration. Using the ddPCR detection method, AAV-2 rss replication rate was identified as ∼3,178-fold in 48 h, measured over an input range from 0.05 to 5,000 vector genomes per well (Fig. 2). If a single infectious event occurs, with 1:50 cell lysate dilution and the use of 5% (1/20th) of the diluted cell lysate, there are ∼3.18 copies per 20 μL PCR, which is about 0.16 copies/μL. Our TCID_50_ data, however, show that the average of negative Ad5-only control value from 18 independent runs is about 0.6 copies/μL (Table 4). Since we omitted the DNA extraction step, 5% (1/20th) of 1:50 diluted crude cell lysate was used directly for PCR; the replicated viral genomes are ∼20 times more diluted compared to the previously published TCID_50_ assay with DNA extraction method.^9^

Therefore, the detection of a single infectious event may not be achievable using our current workflow without DNA extraction. The reduced sensitivity of our current workflow is due to the higher dilution of DNA input to endpoint analysis compared to extraction method.^9^ Given the same amount of DNA input, ddPCR is able to provide more accurate quantitative measurement and higher sensitivity at the lower range from 1.00E+04 to 1.00E+00 copies/μL (Supplementary Fig. S2).

The higher sensitivity observed with ddPCR compared to qPCR may be attributed, in part, to the higher tolerance of PCR inhibitors in ddPCR. The droplet partitioning in ddPCR helps to isolate inhibitors, preventing them from interfering with amplification and improving the accuracy and sensitivity of the assay.^19^ Evaluating the DNA extraction step in TCID_50_ assay is also important because this step adds another layer of assay variability, depending on the consistency of extraction recovery rate between wells and runs. Furthermore, the increased time and cost of the assay with the addition of DNA extraction step will also need to be considered in a manufacturing process.

We also applied statistical analysis to the data points that we generated from the same 18 independent runs, and were able to identify the tolerance intervals of 4 data sets with qPCR or ddPCR from 2 different threshold methods analysis (with or without second set threshold). Interestingly, after applying a second set threshold to analyze the data, we obtained similar tolerant intervals between qPCR (1.25E+08 to 2.81E+09 IU/mL) and ddPCR methods (1.32E+08 to 2.11E+09 IU/mL) (“B” in Table 7). We propose this statistical analysis is important in the future characterization of infectious titer of other AAV serotypes, so that the trend of the data set can be comprehensively monitored and better understood.

While ddPCR presents an improvement in the precision of TCID_50_ analysis, additional work and monitored runs are needed to further optimize controls and thresholding methods for this novel approach. With multiple monitored data points, more appropriate and robust assay acceptance criteria can be obtained for TCID_50_ assay with this newly adopt ddPCR method. For example, the acceptance range of the ddPCR Ad5-only NC and NTC can be set at a value below 3 copies/μL, to ensure that no high background signal can interfere with accurate infectivity scoring toward the low end of detection ranges from higher viral sample dilution group.

Taken together, we have optimized an established cell-based TCID_50_ assay with a novel approach, using state-of-the-art ddPCR system for endpoint analysis. This new approach improves data precision, while simultaneously reducing assay workload by eliminating the need for DNA extraction and standard curve preparation. Together, we aim to contribute to the improvement of rAAV analytical tools to support the increasingly high demands of *in vivo* gene therapies used for disease treatment.

## Supplementary Material

Supplemental data

Supplemental data

Supplemental data

Supplemental data

Supplemental data

Supplemental data

Supplemental data

Supplemental data

Supplemental data

Supplemental data

Supplemental data

Supplemental data

Supplemental data

Supplemental data

Supplemental data

Supplemental data

## Data Availability

All data will be made available upon request.
