# Supplementary material for: Improvement of Precision in Recombinant Adeno-Associated Virus Infectious Titer Assay with Droplet Digital PCR as an Endpoint Measurement
Source: Hum Gene Ther. 2023 Aug 16;34(15-16):742–57. doi: 10.1089/hum.2023.014 (PMC10457655; doi:10.1089/hum.2023.014)
Supplement: Supplemental data [file Supp_TableS2.pdf]

Table S2. AAV2-rss serial dilution scheme

Dilution	Dilution Factor	Viral genome Titer (vg/mL)	Diluent (Ad5) (µL)	AAV2 (µL)	Row in Dilution plate
S2	10^2	1.00E+08	990	10 µL of S1	-
D1	10^4	1.00E+06	990	10 µL of S2	A
D2	10^5	1.00E+05	900	100 µL of D1	B
D3	10^6	1.00E+04	900	100 µL of D2	C
D4	10^7	1.00E+03	900	100 µL of D3	D
D5	10^8	1.00E+02	900	100 µL of D4	E
D6	10^9	1.00E+01	900	100 µL of D5	F
D7	10^{10}	1.00E+00	900	100 µL of D6	G
Negative control	N/A	N/A	1000	N/A	H

Note: First dilution (S1) D = 3.28x; from 3.28E+10 to 1.00E+10
