# Supplementary material for: Improvement of Precision in Recombinant Adeno-Associated Virus Infectious Titer Assay with Droplet Digital PCR as an Endpoint Measurement
Source: Hum Gene Ther. 2023 Aug 16;34(15-16):742–57. doi: 10.1089/hum.2023.014 (PMC10457655; doi:10.1089/hum.2023.014)
Supplement: Supplemental data [file Supp_TableS1.pdf]

Table S1. Infection plate layout

Plate Layout												
	1	2	3	4	5	6	7	8	9	10	11	12
A	D1	D1	D1	D1	D1	D1	D1	D1	D1	D1	Empty	UI
B	D2	D2	D2	D2	D2	D2	D2	D2	D2	D2	Empty	UI
C	D3	D3	D3	D3	D3	D3	D3	D3	D3	D3	Empty	UI
D	D4	D4	D4	D4	D4	D4	D4	D4	D4	D4	Empty	UI
E	D5	D5	D5	D5	D5	D5	D5	D5	D5	D5	Empty	UI
F	D6	D6	D6	D6	D6	D6	D6	D6	D6	D6	Empty	UI
G	D7	D7	D7	D7	D7	D7	D7	D7	D7	D7	Empty	UI
H	NC	NC	NC	NC	NC	NC	NC	NC	NC	NC	Empty	UI

Notes: UI = Uninfected well; NC = Negative Control; D1-D7 = serial AAV dilution
