# Supplementary material for: Improvement of Precision in Recombinant Adeno-Associated Virus Infectious Titer Assay with Droplet Digital PCR as an Endpoint Measurement
Source: Hum Gene Ther. 2023 Aug 16;34(15-16):742–57. doi: 10.1089/hum.2023.014 (PMC10457655; doi:10.1089/hum.2023.014)
Supplement: Supplemental data [file Supp_TableS3.pdf]

Table S3. qPCR and ddPCR plate layout

qPCR Plate Layout												
	1	2	3	4	5	6	7	8	9	10	11	12
A	D1	D1	D1	D1	D1	D1	D1	D1	D1	D1	UI	SD1
B	D2	D2	D2	D2	D2	D2	D2	D2	D2	D2	UI	SD2
C	D3	D3	D3	D3	D3	D3	D3	D3	D3	D3	UI	SD3
D	D4	D4	D4	D4	D4	D4	D4	D4	D4	D4	UI	SD4
E	D5	D5	D5	D5	D5	D5	D5	D5	D5	D5	UI	SD5
F	D6	D6	D6	D6	D6	D6	D6	D6	D6	D6	UI	SD6
G	D7	D7	D7	D7	D7	D7	D7	D7	D7	D7	UI	SD7
H	NC	NC	NC	NC	NC	NC	NC	NC	NC	NC	UI	NTC

ddPCR Plate Layout												
	1	2	3	4	5	6	7	8	9	10	11	12
A	D1	D1	D1	D1	D1	D1	D1	D1	D1	D1	UI	NTC
B	D2	D2	D2	D2	D2	D2	D2	D2	D2	D2	UI	NTC
C	D3	D3	D3	D3	D3	D3	D3	D3	D3	D3	UI	NTC
D	D4	D4	D4	D4	D4	D4	D4	D4	D4	D4	UI	NTC
E	D5	D5	D5	D5	D5	D5	D5	D5	D5	D5	UI	NTC
F	D6	D6	D6	D6	D6	D6	D6	D6	D6	D6	UI	NTC
G	D7	D7	D7	D7	D7	D7	D7	D7	D7	D7	UI	NTC

H	NC	NC	NC	NC	NC	NC	NC	NC	NC	NC	UI	NTC
