# Supplementary material for: Improvement of Precision in Recombinant Adeno-Associated Virus Infectious Titer Assay with Droplet Digital PCR as an Endpoint Measurement
Source: Hum Gene Ther. 2023 Aug 16;34(15-16):742–57. doi: 10.1089/hum.2023.014 (PMC10457655; doi:10.1089/hum.2023.014)
Supplement: Supplemental data [file Supp_TableS12.pdf]

Table S12. Primers and Probe for viral genome detection

	Sequence 5'-3'
CMV_Forward	TACGGTAAACTGCCCACTTG
CMV_Reverse	AGGAAAGTCCCATAAGGTCA
CMV_probe (FAM)	GACGGTAAATGGCCCGCCTG
