# Supplementary material for: Improvement of Precision in Recombinant Adeno-Associated Virus Infectious Titer Assay with Droplet Digital PCR as an Endpoint Measurement
Source: Hum Gene Ther. 2023 Aug 16;34(15-16):742–57. doi: 10.1089/hum.2023.014 (PMC10457655; doi:10.1089/hum.2023.014)
Supplement: Supplemental data [file Supp_TableS8.pdf]

Table S8. Logarithmic transformation of data

	1<br>ID	2<br>qPCR_1	3<br>qPCR_2	4<br>ddPCR_1	5<br>ddPCR_2	6<br>log(qPCR_1)	7<br>log(qPCR_2)	8<br>log(ddPCR_1)	9<br>log(ddPCR_2)
1	Run1-Jan17	1.31E+09	1.31E+09	1.04E+09	6.56E+08	9.1173	9.1173	9.0170	8.8169
2	Run2-Jan31	1.04E+09	1.04E+09	8.26E+08	8.26E+08	9.0170	9.0170	8.9170	8.9169
3	Run3-Feb07	4.14E+08	4.14E+08	8.26E+08	8.26E+08	8.6170	8.6170	8.9170	8.9170
4	Run4-Mar28	4.14E+08	4.14E+08	4.14E+08	3.29E+08	8.6170	8.6170	8.6170	8.5169
5	Run5-Mar31	8.26E+08	8.26E+08	2.07E+09	8.26E+08	8.9170	8.9170	9.3160	8.9169
6	Run6-Apr04	3.29E+08	3.29E+08	3.29E+08	3.29E+08	8.5172	8.5172	8.5172	8.5169
7	Run7-Apr11	4.14E+08	4.14E+08	8.26E+08	3.29E+08	8.6170	8.6170	8.9170	8.5169
8	Run8-Apr18	6.56E+08	6.56E+08	3.29E+08	3.29E+08	8.8169	8.8169	8.5172	8.5169
9	Run9-May09	4.14E+08	4.14E+08	6.56E+08	4.14E+08	8.6170	8.6170	8.8169	8.6169
10	Run10-May16	3.29E+09	6.56E+08	8.26E+08	5.21E+08	9.5169	8.8169	8.9170	8.7169
11	Run11-May23	1.65E+09	8.26E+08	1.65E+09	6.56E+08	9.2169	8.9170	9.2169	8.8169
12	Run12-Jun06	5.21E+08	4.14E+08	4.14E+08	4.14E+08	8.7169	8.6169	8.6169	8.6169
13	Run13-Jun13	5.21E+08	5.21E+08	5.21E+08	5.21E+08	8.7169	8.7169	8.7169	8.7169
14	Run14-Jul05	1.04E+09	1.04E+09	8.26E+08	8.26E+08	9.0169	9.0169	8.9169	8.9169
15	Run15-Nov07	3.29E+08	3.29E+08	3.29E+08	3.29E+08	8.5169	8.5169	8.5169	8.5169
16	Run16-Nov14	5.21E+08	5.21E+08	5.21E+08	5.21E+08	8.7169	8.7169	8.7169	8.7169
17	Run17-Dec02	6.56E+08	6.56E+08	6.56E+08	6.56E+08	8.8169	8.8169	8.8169	8.8169
18	Run18-Dec16	8.26E+08	8.26E+08	8.26E+08	8.26E+08	8.9169	8.9169	8.9169	8.9169
