# Supplementary material for: Improvement of Precision in Recombinant Adeno-Associated Virus Infectious Titer Assay with Droplet Digital PCR as an Endpoint Measurement
Source: Hum Gene Ther. 2023 Aug 16;34(15-16):742–57. doi: 10.1089/hum.2023.014 (PMC10457655; doi:10.1089/hum.2023.014)
Supplement: Supplemental data [file Supp_TableS4.pdf]

Table S4. Identification of detection range of ddPCR method. ddPCR values and infectious scoring table from 1:50 dilution cell lysate

Log Dilution	Replicate 1	Replicate 2	Replicate 3	Replicate 4	Replicate 5	Replicate 6	Replicate 7	Replicate 8	Replicate 9	Replicate 10	Mean
4	8400	10300	1000000	11000	9700	11100	9800	1000000	9200	9000	**207850.00**
5	896	929	678	823	995	867	746	936	891	954	**871.50**
6	116	180	56.4	36.3	140	62.9	45.2	24.8	106	26.4	**79.40**
7	1.4	68.5	17.2	1.9	14.9	2.5	1.1	1.2	1.3	0.6	**11.06**
8	2.1	1	1.3	5.2	2	4.3	1.5	1.7	2	2.6	**2.37**
9	1.9	1.7	1.8	1.3	1	2.1	1.7	2	2	1.4	**1.69**
10	1.5	1.2	1.1	1	1.3	2.1	1.6	0.9	0.7	1.3	**1.27**
(Neg control) Ad only	1.3	2.3	1.4	2.3	2	1.3	1.6	2	2.4	1.8	**1.84**
UI	1.6	1.6	1.3	2	3.7	2.3	1.2	1.5			**1.90**
NTC	1	1	2.2	2.7	1.5	1.4	1.6	2.2			**1.70**
Ad only [avg]	**1.84**										
Ad only [stdev]	**0.42**										
Threshold	**3.11**										

Log Dilution	1	2	3	4	5	6	7	8	9	10	Ratio
4	0.1	0.1	0.1	0.1	0.1	0.1	0.1	0.1	0.1	0.1	**1.0**
5	0.1	0.1	0.1	0.1	0.1	0.1	0.1	0.1	0.1	0.1	**1.0**
6	0.1	0.1	0.1	0.1	0.1	0.1	0.1	0.1	0.1	0.1	**1.0**
7	0.0	0.1	0.1	0.0	0.1	0.0	0.0	0.0	0.0	0.0	**0.3**
8	0.0	0.0	0.0	0.1	0.0	0.1	0.0	0.0	0.0	0.0	**0.2**
9	0.0	0.0	0.0	0.0	0.0	0.0	0.0	0.0	0.0	0.0	**0.0**
10	0.0	0.0	0.0	0.0	0.0	0.0	0.0	0.0	0.0	0.0	**0.0**

**S**	**6.5**
**Infectious Titer (IU/mL)**	**2.00E+08**
**Specific Infectivity (vg/IU)**	**50**
**Adjusted Infectious titer (IU/mL)**	**6.56E+08**
