# Supplementary material for: Improvement of Precision in Recombinant Adeno-Associated Virus Infectious Titer Assay with Droplet Digital PCR as an Endpoint Measurement
Source: Hum Gene Ther. 2023 Aug 16;34(15-16):742–57. doi: 10.1089/hum.2023.014 (PMC10457655; doi:10.1089/hum.2023.014)
Supplement: Supplemental data [file Supp_TableS5.pdf]

Table S5. Identification of detection range of ddPCR method. ddPCR values and infectious scoring table from 1:100 dilution cell lysate.

Log Dilution	Replicate 1	Replicate 2	Replicate 3	Replicate 4	Replicate 5	Replicate 6	Replicate 7	Replicate 8	Replicate 9	Replicate 10	Mean
4	5160	4510	4050	4510	4040	4440	4100	4460	4770	3970	**4401.00**
5	456	459	312	413	474	429	372	441	471	444	**427.10**
6	64.4	98.1	22.6	16.9	70.9	37.5	24.9	13.5	57.1	12.3	**41.82**
7	9.2	32.4	10.2	2	8.4	1.7	1.4	2.2	2.1	2.5	**7.21**
8	3.8	2.1	2	1.6	2	2	1.4	2.2	2.4	1.9	**2.14**
9	2.3	2.1	4	2.3	1.9	2.4	2	2.4	1.8	1.8	**2.30**
10	1.8	2.3	1.7	1.9	1.6	2.3	1.7	2	1.7	2.6	**1.96**
(Neg control) Ad only	1.2	2.3	3	1.6	2.2	2.1	1.8	2.5	1.8	2.2	**2.07**
UI	9.3	2.5	2.1	2.2	2.1	1.7	11.2	1.9			**4.13**
NTC	3.4	2.1	2.9	2.1	1.8	2.7	2.5	2.3			**2.48**
Ad only [avg]	**2.07**										
Ad only [stdev]	**0.50**										
Threshold	**3.57**										

Log Dilution	1	2	3	4	5	6	7	8	9	10	Ratio
4	0.1	0.1	0.1	0.1	0.1	0.1	0.1	0.1	0.1	0.1	**1.0**
5	0.1	0.1	0.1	0.1	0.1	0.1	0.1	0.1	0.1	0.1	**1.0**
6	0.1	0.1	0.1	0.1	0.1	0.1	0.1	0.1	0.1	0.1	**1.0**

**7**	0.1	0.1	0.1	0.0	0.1	0.0	0.0	0.0	0.0	0.0	**0.4**
**8**	0.1	0.0	0.0	0.0	0.0	0.0	0.0	0.0	0.0	0.0	**0.1**
**9**	0.0	0.0	0.1	0.0	0.0	0.0	0.0	0.0	0.0	0.0	**0.1**
**10**	0.0	0.0	0.0	0.0	0.0	0.0	0.0	0.0	0.0	0.0	**0.0**

**S**	**6.6**
**Infectious Titer (IU/mL)**	**2.52E+08**
**Specific Infectivity (vg/IU)**	**40**
**Adjusted Infectious titer (IU/mL)**	**8.26E+08**
