# Supplementary material for: Improvement of Precision in Recombinant Adeno-Associated Virus Infectious Titer Assay with Droplet Digital PCR as an Endpoint Measurement
Source: Hum Gene Ther. 2023 Aug 16;34(15-16):742–57. doi: 10.1089/hum.2023.014 (PMC10457655; doi:10.1089/hum.2023.014)
Supplement: Supplemental data [file Supp_TableS6.pdf]

Table S6. Second set threshold identification is critical for the infectious titer assay with qPCR as an end-point method. **(A, B)** Example run 11 with qPCR analysis. By using average C_T value of Ad5 control minus 3 times of standard deviation (37.47), we got ratios of 0.2; 0.0; 0.1 for the last three dilutions (D5, D6, D7) and the infectious titer at 1.65E+09 (IU/mL). **(C)** By applying the second set threshold as 35 C_T, we got ratios of 0.1; 0.0; 0.0 for the last three dilution (D5, D6, D7) and the infectious titer at 8.26E+08 (IU/mL).

A.

Log Dilution	Replicate 1	Replicate 2	Replicate 3	Replicate 4	Replicate 5	Replicate 6	Replicate 7	Replicate 8	Replicate 9	Replicate 10	Mean
4	21.64	22.72	21.98	21.85	22.13	22.13	22.09	21.51	21.74	21.2	**21.90**
5	25.9	25.5	25.78	25.68	25.57	24.82	26.05	25.25	26.05	23.43	**25.40**
6	27.99	29.39	28.49	30.68	30.25	30.22	29.76	28.29	29.79	28.93	**29.38**
7	40	32.86	34.39	30.34	38.06	40	35.82	40	30.84	29.91	**35.22**
8	36.53	40	40	40	40	40	40	31.24	40	38.18	**38.60**
9	38.71	40	38.49	40	40	38.57	40	40	40	40	**39.58**
10	40	40	40	40	40	39.6	37.21	40	40	40	**39.68**
(Neg control) Ad only	39.15	40	38.32	40	40	40	40	40	40	38.37	**39.58**
UI	40	40	38.46	40	40	40	40	40			**39.81**
NTC	40										

Ad only [avg]	**39.58**
Ad only [stdev]	**0.70**
Threshold Ct	**37.47**

B.

Log Dilution	1	2	3	4	5	6	7	8	9	10	Ratio
4	0.1	0.1	0.1	0.1	0.1	0.1	0.1	0.1	0.1	0.1	**1.0**
5	0.1	0.1	0.1	0.1	0.1	0.1	0.1	0.1	0.1	0.1	**1.0**
6	0.1	0.1	0.1	0.1	0.1	0.1	0.1	0.1	0.1	0.1	**1.0**
7	0.0	0.1	0.1	0.1	0.0	0.0	0.1	0.0	0.1	0.1	**0.6**
8	0.1	0.0	0.0	0.0	0.0	0.0	0.0	0.1	0.0	0.0	**0.2**
9	0.0	0.0	0.0	0.0	0.0	0.0	0.0	0.0	0.0	0.0	**0.0**
10	0.0	0.0	0.0	0.0	0.0	0.0	0.1	0.0	0.0	0.0	**0.1**

S	**6.9**
Infectious Titer (IU/mL)	**5.02E+08**
Specific Infectivity (vg/IU)	**20**
Adjusted Infectious titer (IU/mL)	**1.65E+09**

C.

Ad only [avg]	**39.58**
Ad only [stdev]	**0.70**
Threshold Ct	**37.47**
Set Threshold	**35.00**

Log Dilution	1	2	3	4	5	6	7	8	9	10	Ratio
**4**	0.1	0.1	0.1	0.1	0.1	0.1	0.1	0.1	0.1	0.1	**1.0**
**5**	0.1	0.1	0.1	0.1	0.1	0.1	0.1	0.1	0.1	0.1	**1.0**
**6**	0.1	0.1	0.1	0.1	0.1	0.1	0.1	0.1	0.1	0.1	**1.0**
**7**	0.0	0.1	0.1	0.1	0.0	0.0	0.0	0.0	0.1	0.1	**0.5**
**8**	0.0	0.0	0.0	0.0	0.0	0.0	0.0	0.1	0.0	0.0	**0.1**
**9**	0.0	0.0	0.0	0.0	0.0	0.0	0.0	0.0	0.0	0.0	**0.0**
**10**	0.0	0.0	0.0	0.0	0.0	0.0	0.0	0.0	0.0	0.0	**0.0**

**S**	**6.6**
**Infectious Titer (IU/mL)**	**2.52E+08**
**Specific Infectivity (vg/IU)**	**40**
**Adjusted Infectious titer (IU/mL)**	**8.26E+08**
