# Supplementary material for: Improvement of Precision in Recombinant Adeno-Associated Virus Infectious Titer Assay with Droplet Digital PCR as an Endpoint Measurement
Source: Hum Gene Ther. 2023 Aug 16;34(15-16):742–57. doi: 10.1089/hum.2023.014 (PMC10457655; doi:10.1089/hum.2023.014)
Supplement: Supplemental data [file Supp_TableS7.pdf]

Table S7. Set threshold identification is critical for infectious titer assay with adapted ddPCR as an end-point method. **(A, B)** Example run 5 with ddPCR end-point method that had issue with original Ad5 average threshold. The average copy number value for Ad5-only-infected wells was 0.15 with a standard deviation (stdev) of 0.07. The threshold copy number for this run is calculated as Ad5-only average plus 3 stdev (0.35). By applying this value as a threshold, we got ratios of 0.1; 0.2; 0.1 for the last three dilutions (D5, D6, D7) and the infectious titer at 2.07E+09 (IU/mL). **(C)** By applying an additional of set threshold as 1.5, we got the ratio of 0.1; 0.0; 0.0 for the last three dilutions (D5, D6, D7) and the infectious titer at 8.26E+08 (IU/mL).

A.

Log Dilution	Replicate 1	Replicate 2	Replicate 3	Replicate 4	Replicate 5	Replicate 6	Replicate 7	Replicate 8	Replicate 9	Replicate 10	Mean
4	3230	4770	3920	4610	3830	4420	4120	4450	4260	3760	**4137.00**
5	183	286	300	440	326	341	356	303	289	352	**317.60**
6	12.3	13.6	53.8	17.3	8	42.1	32	48.8	12	69.5	**30.94**
7	0.12	0.19	0.82	0	0.06	2.3	5	42.1	19.1	4.1	**7.38**
8	0.13	0.19	0.33	0.33	0.06	16.6	0.26	0.07	0.06	0	**1.80**
9	0.12	0.19	0.13	0.58	0.44	0.32	0.14	0.34	0.06	0.07	**0.24**
10	0.13	0	0.19	0.07	0.56	0.19	0.33	0.19	0.19	0.06	**0.19**
(Neg control) Ad only	0.13	0.06	0.13	0.2	0.25	0.13	0.06	0.13	0.25	0.18	**0.15**
UI	0.07	0	0.19	0.14	0.07	0.06	0	0			**0.07**
NTC	0.15	0.14	0.29	0.17	0.07	0.16	0.08	0.42			**0.19**

Ad only [avg]	**0.15**
Ad only [stdev]	**0.07**
Threshold	**0.35**

B.

Log Dilution	1	2	3	4	5	6	7	8	9	10	Ratio
4	0.1	0.1	0.1	0.1	0.1	0.1	0.1	0.1	0.1	0.1	**1.0**
5	0.1	0.1	0.1	0.1	0.1	0.1	0.1	0.1	0.1	0.1	**1.0**
6	0.1	0.1	0.1	0.1	0.1	0.1	0.1	0.1	0.1	0.1	**1.0**
7	0.0	0.0	0.1	0.0	0.0	0.1	0.1	0.1	0.1	0.1	**0.6**
8	0.0	0.0	0.0	0.0	0.0	0.1	0.0	0.0	0.0	0.0	**0.1**
9	0.0	0.0	0.0	0.1	0.1	0.0	0.0	0.0	0.0	0.0	**0.2**
10	0.0	0.0	0.0	0.0	0.1	0.0	0.0	0.0	0.0	0.0	**0.1**

S	7.0

**Infectious Titer (IU/mL)**	6.32E+08
**Specific Infectivity (vg/IU)**	16
**Adjusted Infectious titer (IU/mL)**	2.07E+09

C.

**Ad only [avg]**	**0.15**
**Ad only [stdev]**	**0.07**
**Threshold**	**0.35**
**Set Threshold**	**1.70**

Log Dilution	1	2	3	4	5	6	7	8	9	10	Ratio
**4**	0.1	0.1	0.1	0.1	0.1	0.1	0.1	0.1	0.1	0.1	**1.0**
**5**	0.1	0.1	0.1	0.1	0.1	0.1	0.1	0.1	0.1	0.1	**1.0**
**6**	0.1	0.1	0.1	0.1	0.1	0.1	0.1	0.1	0.1	0.1	**1.0**
**7**	0.0	0.0	0.0	0.0	0.0	0.1	0.1	0.1	0.1	0.1	**0.5**
**8**	0.0	0.0	0.0	0.0	0.0	0.1	0.0	0.0	0.0	0.0	**0.1**
**9**	0.0	0.0	0.0	0.0	0.0	0.0	0.0	0.0	0.0	0.0	**0.0**
**10**	0.0	0.0	0.0	0.0	0.0	0.0	0.0	0.0	0.0	0.0	**0.0**

**S**	6.6
**Infectious Titer (IU/mL)**	2.52E+08
**Specific Infectivity (vg/IU)**	40
**Adjusted Infectious titer (IU/mL)**	8.26E+08
