# Supplementary material for: Improvement of Precision in Recombinant Adeno-Associated Virus Infectious Titer Assay with Droplet Digital PCR as an Endpoint Measurement
Source: Hum Gene Ther. 2023 Aug 16;34(15-16):742–57. doi: 10.1089/hum.2023.014 (PMC10457655; doi:10.1089/hum.2023.014)
Supplement: Supplemental data [file Supp_TableS13.pdf]

Table S13. TCID$_{50}$ qPCR runs with "false positive" wells in the highest dilution D7 group (Run 10 and Run 11). (A) In run 10, two "false positive" wells at highest dilution group have high Ct value (37.22 and 37.24, highlighted) which are close to Threshold Ct at 37.73. (B) In run 11, one "false positive" wells at highest dilution group has a high Ct value (37.21, highlighted) which are close to Threshold Ct at 37.47.

A.

Log Dilution	Replicate 1	Replicate 2	Replicate 3	Replicate 4	Replicate 5	Replicate 6	Replicate 7	Replicate 8	Replicate 9	Replicate 10	Mean
4	19.99	18.87	20.29	19.86	19.27	19.56	19.27	20.25	19.68	20.24	**19.73**
5	23.65	23.59	23.92	23.2	24.61	23.25	23.79	22.98	23.33	23.74	**23.61**
6	25.99	26.85	26.88	26.32	28.81	27.9	24.35	30.16	26.13	25.91	**26.93**
7	32.6	29.22	37.3	38.39	40	40	40	40	27.19	28.86	**35.36**
8	40	40	37.3	36.86	30.28	38.51	40	40	38.33	40	**38.13**
9	40	38.28	40	40	40	40	40	38.56	37.3	37.29	**39.14**
10	**37.22**	40	40	**37.24**	40	40	40	40	40	38.46	**39.29**
(Neg control) Ad only	40	40	40	40	40	40	37.84	40	40	40	**39.78**
UI	38.26	36.79	40	40	40	38.35					**38.90**
NTC	40										

Ad only [avg]	**39.78**
Ad only [stdev]	**0.68**
Threshold Ct	**37.73**

Log Dilution	1	2	3	4	5	6	7	8	9	10	Ratio
4	0.1	0.1	0.1	0.1	0.1	0.1	0.1	0.1	0.1	0.1	**1.0**
5	0.1	0.1	0.1	0.1	0.1	0.1	0.1	0.1	0.1	0.1	**1.0**
6	0.1	0.1	0.1	0.1	0.1	0.1	0.1	0.1	0.1	0.1	**1.0**
7	0.1	0.1	0.1	0.0	0.0	0.0	0.0	0.0	0.1	0.1	**0.5**
8	0.0	0.0	0.1	0.1	0.1	0.0	0.0	0.0	0.0	0.0	**0.3**
9	0.0	0.0	0.0	0.0	0.0	0.0	0.0	0.0	0.1	0.1	**0.2**
10	**0.1**	0.0	0.0	**0.1**	0.0	0.0	0.0	0.0	0.0	0.0	**0.2**

B.

Log Dilution	Replicate 1	Replicate 2	Replicate 3	Replicate 4	Replicate 5	Replicate 6	Replicate 7	Replicate 8	Replicate 9	Replicate 10	Mean
4	21.64	22.72	21.98	21.85	22.13	22.13	22.09	21.51	21.74	21.2	**21.90**
5	25.9	25.5	25.78	25.68	25.57	24.82	26.05	25.25	26.05	23.43	**25.40**
6	27.99	29.39	28.49	30.68	30.25	30.22	29.76	28.29	29.79	28.93	**29.38**
7	40	32.86	34.39	30.34	38.06	40	35.82	40	30.84	29.91	**35.22**
8	36.53	40	40	40	40	40	40	31.24	40	38.18	**38.60**
9	38.71	40	38.49	40	40	38.57	40	40	40	40	**39.58**
10	40	40	40	40	40	39.6	**37.21**	40	40	40	**39.68**
(Neg control) Ad only	39.15	40	38.32	40	40	40	40	40	40	38.37	**39.58**
UI	40	40	38.46	40	40	40	40	40			**39.81**
NTC	40										

Ad only [avg]	**39.58**
Ad only [stdev]	**0.70**
Threshold Ct	**37.47**

Log Dilution	1	2	3	4	5	6	7	8	9	10	Ratio
4	0.1	0.1	0.1	0.1	0.1	0.1	0.1	0.1	0.1	0.1	**1.0**
5	0.1	0.1	0.1	0.1	0.1	0.1	0.1	0.1	0.1	0.1	**1.0**
6	0.1	0.1	0.1	0.1	0.1	0.1	0.1	0.1	0.1	0.1	**1.0**
7	0.0	0.1	0.1	0.1	0.0	0.0	0.1	0.0	0.1	0.1	**0.6**
8	0.1	0.0	0.0	0.0	0.0	0.0	0.0	0.1	0.0	0.0	**0.2**
9	0.0	0.0	0.0	0.0	0.0	0.0	0.0	0.0	0.0	0.0	**0.0**
10	0.0	0.0	0.0	0.0	0.0	0.0	**0.1**	0.0	0.0	0.0	**0.1**
