# Supplementary material for: Improvement of Precision in Recombinant Adeno-Associated Virus Infectious Titer Assay with Droplet Digital PCR as an Endpoint Measurement
Source: Hum Gene Ther. 2023 Aug 16;34(15-16):742–57. doi: 10.1089/hum.2023.014 (PMC10457655; doi:10.1089/hum.2023.014)
Supplement: Supplemental data [file Supp_TableS14.pdf]

Table S14. $TCID_{50}$ ddPCR Runs with "false positive" wells in the highest dilution D7 group (Run 5 and Run 11). (A) In run 5, one "false positive" well at highest dilution group have low concentration (copies/µL) value (0.56, highlighted) which are close to Threshold concentration at 0.35. (B) In run 11, one "false positive" wells at highest dilution group has a low concentration (copies/µL) value (1.1, highlighted) which are close to Threshold concentration at 0.35.

A.

Log Dilution	Replicate 1	Replicate 2	Replicate 3	Replicate 4	Replicate 5	Replicate 6	Replicate 7	Replicate 8	Replicate 9	Replicate 10	Mean
4	3230	4770	3920	4610	3830	4420	4120	4450	4260	3760	**4137.00**
5	183	286	300	440	326	341	356	303	289	352	**317.60**
6	12.3	13.6	53.8	17.3	8	42.1	32	48.8	12	69.5	**30.94**
7	0.12	0.19	0.82	0	0.06	2.3	5	42.1	19.1	4.1	**7.38**
8	0.13	0.19	0.33	0.33	0.06	16.6	0.26	0.07	0.06	0	**1.80**
9	0.12	0.19	0.13	0.58	0.44	0.32	0.14	0.34	0.06	0.07	**0.24**
10	0.13	0	0.19	0.07	**0.56**	0.19	0.33	0.19	0.19	0.06	**0.19**
(Neg control) Ad only	0.13	0.06	0.13	0.2	0.25	0.13	0.06	0.13	0.25	0.18	**0.15**
UI	0.07	0	0.19	0.14	0.07	0.06	0	0			**0.07**
NTC	0.15	0.14	0.29	0.17	0.07	0.16	0.08	0.42			**0.19**
Ad only [avg]	**0.15**										
Ad only [stdev]	**0.07**										
Threshold	**0.35**										

Log Dilution	1	2	3	4	5	6	7	8	9	10	Ratio
4	0.1	0.1	0.1	0.1	0.1	0.1	0.1	0.1	0.1	0.1	**1.0**
5	0.1	0.1	0.1	0.1	0.1	0.1	0.1	0.1	0.1	0.1	**1.0**
6	0.1	0.1	0.1	0.1	0.1	0.1	0.1	0.1	0.1	0.1	**1.0**
7	0.0	0.0	0.1	0.0	0.0	0.1	0.1	0.1	0.1	0.1	**0.6**
8	0.0	0.0	0.0	0.0	0.0	0.1	0.0	0.0	0.0	0.0	**0.1**
9	0.0	0.0	0.0	0.1	0.1	0.0	0.0	0.0	0.0	0.0	**0.2**
10	0.0	0.0	0.0	0.0	**0.1**	0.0	0.0	0.0	0.0	0.0	**0.1**

B.

Log Dilution	Replicate 1	Replicate 2	Replicate 3	Replicate 4	Replicate 5	Replicate 6	Replicate 7	Replicate 8	Replicate 9	Replicate 10	Mean
4	10000	10000	8000	10200	8200	9100	8300	8600	10300	9800	**9250.00**
5	581	823	587	708	704	1125	569	1008	646	921	**767.20**
6	153	55.9	121	26.2	34.4	29.3	44.5	107	46.5	84	**70.18**
7	0	6.4	0.9	26.6	0.8	0.17	0.65	0.06	26.4	44.7	**10.67**
8	0	0.11	0	0.19	0	0.18	0.07	21.4	0.14	0.08	**2.22**
9	0.24	0.33	0	0.09	0	0	0	0	0.07	0.08	**0.08**
10	0.11	**1.1**	0	0.1	0	0.21	0.16	0.3	0	0.09	**0.21**
(Neg control) Ad only	0	0.11	0	0	0.1	0	0.09	0.18	0.24	0.17	**0.09**
UI	0.09	0.18	0	0.48	0.08	0	0.08	0			**0.11**
NTC	0.11										
Ad only [avg]	**0.09**										
Ad only [stdev]	**0.09**										
Threshold	**0.35**										

Log Dilution	1	2	3	4	5	6	7	8	9	10	Ratio
4	0.1	0.1	0.1	0.1	0.1	0.1	0.1	0.1	0.1	0.1	**1.0**
5	0.1	0.1	0.1	0.1	0.1	0.1	0.1	0.1	0.1	0.1	**1.0**
6	0.1	0.1	0.1	0.1	0.1	0.1	0.1	0.1	0.1	0.1	**1.0**
7	0.0	0.1	0.1	0.1	0.1	0.0	0.1	0.0	0.1	0.1	**0.7**
8	0.0	0.0	0.0	0.0	0.0	0.0	0.0	0.1	0.0	0.0	**0.1**
9	0.0	0.0	0.0	0.0	0.0	0.0	0.0	0.0	0.0	0.0	**0.0**
10	0.0	**0.1**	0.0	0.0	0.0	0.0	0.0	0.0	0.0	0.0	**0.1**
