# Supplementary material for: Improvement of Precision in Recombinant Adeno-Associated Virus Infectious Titer Assay with Droplet Digital PCR as an Endpoint Measurement
Source: Hum Gene Ther. 2023 Aug 16;34(15-16):742–57. doi: 10.1089/hum.2023.014 (PMC10457655; doi:10.1089/hum.2023.014)

Supplemental Materials

Supplemental Figure:

Figure S1. Summary of 18 independent TCID$_{50}$ runs analyzed with qPCR _1 (A), qPCR_2 (B), ddPCR_1 (C), ddPCR_2 (D). A trending lines were added to each graph to evaluate infectious titer trend over one year testing period. Statistical analysis on the trending of infectious value over time identified no significant downward trend in infectious titer during one year study.

Figure S1.

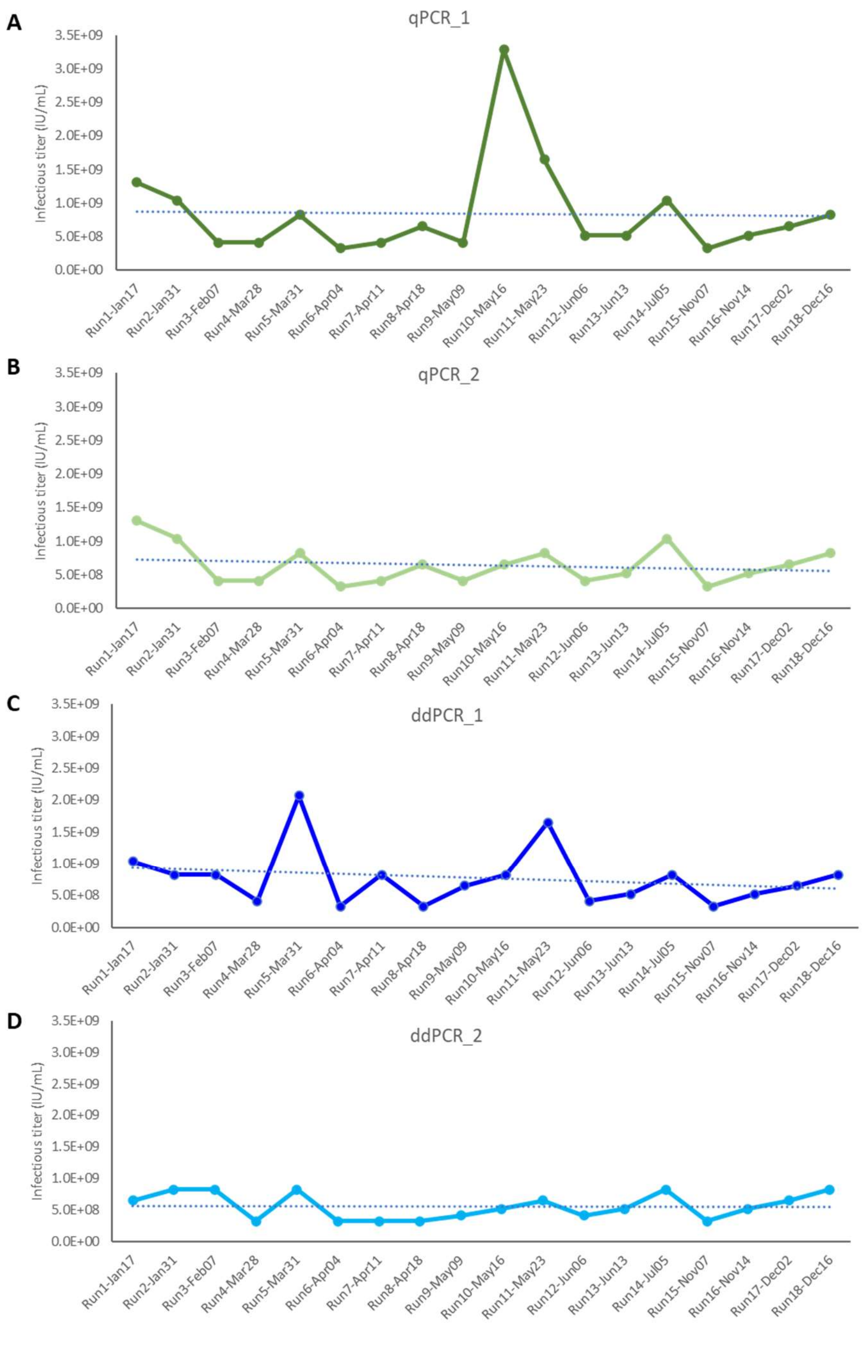

Supplement: Supplemental data [file Supp_FigS1.pdf]
