# Supplementary material for: Improvement of Precision in Recombinant Adeno-Associated Virus Infectious Titer Assay with Droplet Digital PCR as an Endpoint Measurement
Source: Hum Gene Ther. 2023 Aug 16;34(15-16):742–57. doi: 10.1089/hum.2023.014 (PMC10457655; doi:10.1089/hum.2023.014)
Supplement: Supplemental data [file Supp_TableS11.pdf]

Table S11. Calculation of Tolerance Intervals

Log(qPCR_1)

	Normal Tolerance Limits
	Value
Number of Cases	18
Mean	8.83364
Sigma	.266228
Lower Specification Limit	8.56744
Nominal Specification	8.83364
Upper Specification Limit	9.09984
Confidence Level	95.0000%
% of Population Included	99.0000%
Lower Interval Limit	7.84681
Upper Interval Limit	9.82047

Log(qPCR_2)

	Normal Tolerance Limits
	Value
Number of Cases	18
Mean	8.77253
Sigma	.182231
Lower Specification Limit	8.59030
Nominal Specification	8.77250
Upper Specification Limit	8.95470
Confidence Level	95.0000%
% of Population Included	99.0000%
Lower Interval Limit	8.09706
Upper Interval Limit	9.44801

Log(ddPCR_1)

	Normal Tolerance Limits
	Value
Number of Cases	18
Mean	8.82802
Sigma	.227074
Lower Specification Limit	8.60090
Nominal Specification	8.82800
Upper Specification Limit	9.05510
Confidence Level	95.0000%
% of Population Included	99.0000%
Lower Interval Limit	7.98633
Upper Interval Limit	9.66972

Log(ddPCR_2)

	Normal Tolerance Limits
	Value
Number of Cases	18
Mean	8.72246
Sigma	.162603
Lower Specification Limit	8.55990
Nominal Specification	8.72250
Upper Specification Limit	8.88510
Confidence Level	95.0000%
% of Population Included	99.0000%
Lower Interval Limit	8.11974
Upper Interval Limit	9.32518
