# Supplementary material for: Improvement of Precision in Recombinant Adeno-Associated Virus Infectious Titer Assay with Droplet Digital PCR as an Endpoint Measurement
Source: Hum Gene Ther. 2023 Aug 16;34(15-16):742–57. doi: 10.1089/hum.2023.014 (PMC10457655; doi:10.1089/hum.2023.014)
Supplement: Supplemental data [file Supp_TableS9.pdf]

Table S9. Check for potential outliers

Variable	Descriptive Statistics (TCID50 qPCR ddPCR 29Mar23 in qPCR ddPCR Summary)						
	Valid N	Mean	Grubbs Test Statistic	p-value	Minimum	Maximum	Std.Dev.
log(qPCR_1)	18	8.833640	2.566460	0.075380	8.516904	9.516904	0.266228
log(qPCR_2)	18	8.772533	1.891769	0.862162	8.516904	9.117271	0.182231
log(ddPCR_1)	18	8.828025	2.148837	0.391946	8.516904	9.315970	0.227074
log(ddPCR_2)	18	8.722464	1.264184	1.000000	8.516904	8.916980	0.162603
