# Supplementary material for: Improvement of Precision in Recombinant Adeno-Associated Virus Infectious Titer Assay with Droplet Digital PCR as an Endpoint Measurement
Source: Hum Gene Ther. 2023 Aug 16;34(15-16):742–57. doi: 10.1089/hum.2023.014 (PMC10457655; doi:10.1089/hum.2023.014)
Supplement: Supplemental data [file Supp_TableS10.pdf]

Table S10. Check for Normal Distribution

Log(qPCR_1)

Parameter estimates for all distributions (TCID50 qPCR ddPCR 29Mar23 in qPCR ddPCR Summary)
Variable:log(qPCR_1) N = 18
(p-values assume a-priori knowledge of parameters)
The distributions are ordered (from the top) in terms of how well they fit the data.
All distributions with the p-value for the Kolmogorov-Smirnov test statistics that are either (in order of best to worst) n.s, 0.1<= p < 0.2 or 0.05<=p<0.1 are suitable models for the data.
Note: If Normal (location, scale) is among the suitable models use the Normal (location, scale)

Distribution	User-def Param 1	User-def Param 2	Param 1	Param 2	K-S d	**K-S p-value**
Non-Normal (skewness, kurtosis)			1.061446	1.02305	0.112324	n.s.
Extreme Value (location, scale)			8.717327	0.19090	0.149069	n.s.
Log-Normal (threshold, scale, shape)	0.00		2.178146	0.02974	0.167531	n.s.
**Normal (location, scale)**			8.833640	0.26623	0.169481	n.s.
Weibull (threshold, scale, shape)	0.00		8.968468	30.05305	0.190790	n.s.
Rayleigh (threshold, scale)	0.00		6.249005		0.604964	p<.01
Exponential (threshold, scale)	0.00		8.833640		0.618691	p<.01
Beta (threshold, sigma, shape, shape)	0.00	1.000000				--
Gamma (threshold, scale, shape)	0.00					--

Log(qPCR_2)

Parameter estimates for all distributions (TCID50 qPCR ddPCR 29Mar23 in qPCR ddPCR Summary)
Variable:log(qPCR_2) N = 18
(p-values assume a-priori knowledge of parameters)
The distributions are ordered (from the top) in terms of how well they fit the data.
All distributions with the p-value for the Kolmogorov-Smirnov test statistics that are either (in order of best to worst) n.s, 0.1<= p < 0.2 or 0.05<=p<0.1 are suitable models for the data.
Note: If Normal (location, scale) is among the suitable models use the Normal (location, scale)

Distribution	User-def Param 1	User-def Param 2	Param 1	Param 2	K-S d	**K-S p-value**
Non-Normal (skewness, kurtosis)			0.294860	-1.02446	0.142811	n.s.
Weibull (threshold, scale, shape)	0.00		8.861159	51.41909	0.177315	n.s.
Extreme Value (location, scale)			8.686807	0.14976	0.185735	n.s.
Log-Normal (threshold, scale, shape)	0.00		2.171422	0.02072	0.192181	n.s.
**Normal (location, scale)**			8.772533	0.18223	0.192196	n.s.
Rayleigh (threshold, scale)	0.00		6.204381		0.610225	p<.01
Exponential (threshold, scale)	0.00		8.772533		0.621243	p<.01
Beta (threshold, sigma, shape, shape)	0.00	1.000000				--
Gamma (threshold, scale, shape)	0.00					--

Log(ddPCR_1)

Parameter estimates for all distributions (TCID50 qPCR ddPCR 29Mar23 in qPCR ddPCR Summary)
Variable:log(ddPCR_1) N = 18
(p-values assume a-priori knowledge of parameters)
The distributions are ordered (from the top) in terms of how well they fit the data.
All distributions with the p-value for the Kolmogorov-Smirnov test statistics that are either (in order of best to worst) n.s, 0.1<= p < 0.2 or 0.05<=p<0.1 are suitable models for the data.
Note: If Normal (location, scale) is among the suitable models use the Normal (location, scale)

Distribution	User-def Param 1	User-def Param 2	Param 1	Param 2	K-S d	**K-S p-value**
Non-Normal (skewness, kurtosis)			0.419518	-0.03341	0.171382	n.s.
Log-Normal (threshold, scale, shape)	0.00		2.177621	0.02562	0.176612	n.s.
**Normal (location, scale)**			8.828025	0.22707	0.180957	n.s.
Extreme Value (location, scale)			8.721648	0.19027	0.198814	n.s.
Weibull (threshold, scale, shape)	0.00		8.939058	38.96220	0.236604	n.s.
Rayleigh (threshold, scale)	0.00		6.244307		0.605516	p<.01
Exponential (threshold, scale)	0.00		8.828025		0.618924	p<.01
Beta (threshold, sigma, shape, shape)	0.00	1.000000				--
Gamma (threshold, scale, shape)	0.00					--

Log(ddPCR_2)

Parameter estimates for all distributions (TCID50 qPCR ddPCR 29Mar23 in qPCR ddPCR Summary)
Variable:log(ddPCR_2) N = 18
(p-values assume a-priori knowledge of parameters)
The distributions are ordered (from the top) in terms of how well they fit the data.
All distributions with the p-value for the Kolmogorov-Smirnov test statistics that are either (in order of best to worst) n.s, 0.1<= p < 0.2 or 0.05<=p<0.1 are suitable models for the data.
Note: If Normal (location, scale) is among the suitable models use the Normal (location, scale)

Distribution	User-def Param 1	User-def Param 2	Param 1	Param 2	K-S d	**K-S p-value**
Non-Normal (skewness, kurtosis)			-0.099914	-1.62639	0.162736	n.s.
**Normal (location, scale)**			8.722464	0.16260	0.174696	n.s.
Log-Normal (threshold, scale, shape)	0.00		2.165737	0.01866	0.175597	n.s.
Weibull (threshold, scale, shape)	0.00		8.799037	64.63262	0.183662	n.s.
Extreme Value (location, scale)			8.643253	0.14160	0.190674	n.s.
Rayleigh (threshold, scale)	0.00		6.168725		0.614460	p<.01
Exponential (threshold, scale)	0.00		8.722464		0.623348	p<.01
Beta (threshold, sigma, shape, shape)	0.00	1.000000				--
Gamma (threshold, scale, shape)	0.00					--
