# Supplementary material for: Improvement of Precision in Recombinant Adeno-Associated Virus Infectious Titer Assay with Droplet Digital PCR as an Endpoint Measurement
Source: Hum Gene Ther. 2023 Aug 16;34(15-16):742–57. doi: 10.1089/hum.2023.014 (PMC10457655; doi:10.1089/hum.2023.014)
Supplement: Supplemental data [file Supp_FigS2.pdf]

Figure S2. Limit of quantification (LOQ) experiment for qPCR and ddPCR method. (A) Graph shows the comparison between input viral genome concentration with ddPCR and qPCR value. Inputs are AAV2 rss viral genome in 10x serial dilution from 1.00E+04 to 2.00E-01 (copies/µL). **(B)** Table shows the viral genome concentration (copies/µL) of input, ddPCR and qPCR output.

Figure S2.

A

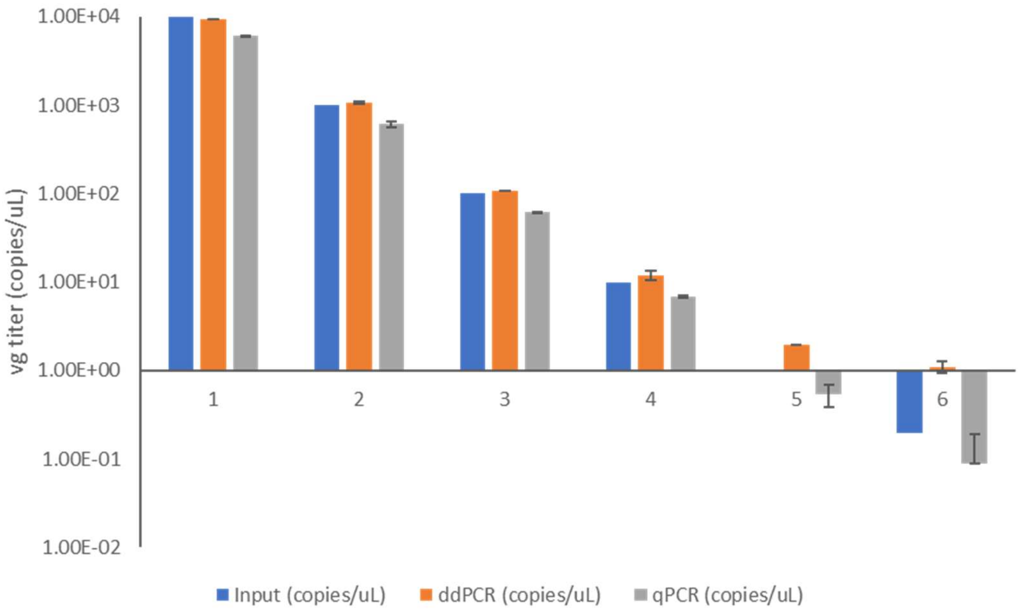

B

	Input (copies/uL)	ddPCR (copies/uL)	qPCR (copies/uL)
1	1.00E+04	9.37E+03	6.07E+03
2	1.00E+03	1.07E+03	6.11E+02
3	1.00E+02	1.08E+02	6.11E+01
4	1.00E+01	1.20E+01	6.85E+00
5	1.00E+00	1.95E+00	5.42E-01
6	2.00E-01	1.11E+00	8.88E-02
